# Oncogenic PIK3CA mutations shape an immunoregulatory microenvironment in mosaic overgrowth disorders

**DOI:** 10.1093/pnasnexus/pgag163

**Published:** 2026-05-13

**Authors:** Ilaria Galasso, Charles Bayard, Camille Blériot, Sophia Ladraa, Wan Ting Kong, Rubina Cassaca, Clément Hoguin, Sanela Protic, Jérôme Megret, Nicolas Goudin, Nicolas Signolle, Jean-Yves Scoazec, Ivan Nemazanyy, Estelle Balducci, Patrick Villarese, Vahid Asnafi, Sylvie Fraitag, Nicolas Venteclef, Florent Ginhoux, Guillaume Canaud

**Affiliations:** Université Paris Cité, 75006 Paris, France; Unité de Médecine Translationnelle et Thérapies Ciblées, Hôpital Necker-Enfants Malades, AP-HP, 75015 Paris, France; Institut National de la Santé et de la Recherche Médicale U1151, Institut Necker-Enfants Malades, 149 rue de Sèvres, 75015 Paris, France; Université Paris Cité, 75006 Paris, France; Unité de Médecine Translationnelle et Thérapies Ciblées, Hôpital Necker-Enfants Malades, AP-HP, 75015 Paris, France; Institut National de la Santé et de la Recherche Médicale U1151, Institut Necker-Enfants Malades, 149 rue de Sèvres, 75015 Paris, France; Institut National de la Santé et de la Recherche Médicale U1151, Institut Necker-Enfants Malades, 149 rue de Sèvres, 75015 Paris, France; INSERM U1015, Gustave Roussy Cancer Campus, 94085 Villejuif, France; Université Paris Cité, 75006 Paris, France; Unité de Médecine Translationnelle et Thérapies Ciblées, Hôpital Necker-Enfants Malades, AP-HP, 75015 Paris, France; Institut National de la Santé et de la Recherche Médicale U1151, Institut Necker-Enfants Malades, 149 rue de Sèvres, 75015 Paris, France; INSERM U1015, Gustave Roussy Cancer Campus, 94085 Villejuif, France; Université Paris Cité, 75006 Paris, France; Unité de Médecine Translationnelle et Thérapies Ciblées, Hôpital Necker-Enfants Malades, AP-HP, 75015 Paris, France; Institut National de la Santé et de la Recherche Médicale U1151, Institut Necker-Enfants Malades, 149 rue de Sèvres, 75015 Paris, France; Université Paris Cité, 75006 Paris, France; Unité de Médecine Translationnelle et Thérapies Ciblées, Hôpital Necker-Enfants Malades, AP-HP, 75015 Paris, France; Institut National de la Santé et de la Recherche Médicale U1151, Institut Necker-Enfants Malades, 149 rue de Sèvres, 75015 Paris, France; Université Paris Cité, 75006 Paris, France; Unité de Médecine Translationnelle et Thérapies Ciblées, Hôpital Necker-Enfants Malades, AP-HP, 75015 Paris, France; Institut National de la Santé et de la Recherche Médicale U1151, Institut Necker-Enfants Malades, 149 rue de Sèvres, 75015 Paris, France; Structure Fédérative de Recherche Necker, INSERM US24, CNRS UAR 3633, Institut Necker-Enfants Malades, 75015 Paris, France; Necker Bio-Image Analysis, INSERM US24/CNRS UMS 3633, 75015 Paris, France; Biopathology Department, Gustave Roussy Cancer Campus, 94085 Villejuif, France; Département de Biologie et Pathologie Médicale, Gustave Roussy Cancer Campus, 94085 Villejuif, France; Platform for Metabolic Analyses, Structure Fédérative de Recherche Necker, INSERM US24, CNRS UAR 3633, Institut Necker-Enfants Malades, 94085 Paris, France; Laboratoire d’oncologie Hématologie, Hôpital Necker-Enfants Malades, AP-HP, 75015 Paris, France; Laboratoire d’oncologie Hématologie, Hôpital Necker-Enfants Malades, AP-HP, 75015 Paris, France; Université Paris Cité, 75006 Paris, France; Institut National de la Santé et de la Recherche Médicale U1151, Institut Necker-Enfants Malades, 149 rue de Sèvres, 75015 Paris, France; Laboratoire d’oncologie Hématologie, Hôpital Necker-Enfants Malades, AP-HP, 75015 Paris, France; Service d’Anatomie Pathologique, Hôpital Necker-Enfants Malades, AP-HP, 75015 Paris, France; Institut National de la Santé et de la Recherche Médicale U1151, Institut Necker-Enfants Malades, 149 rue de Sèvres, 75015 Paris, France; INSERM U1015, Gustave Roussy Cancer Campus, 94085 Villejuif, France; Université Paris Cité, 75006 Paris, France; Unité de Médecine Translationnelle et Thérapies Ciblées, Hôpital Necker-Enfants Malades, AP-HP, 75015 Paris, France; Institut National de la Santé et de la Recherche Médicale U1151, Institut Necker-Enfants Malades, 149 rue de Sèvres, 75015 Paris, France

**Keywords:** PIK3CA-related overgrowth syndrome, Warburg like Tumor microenvironment, macrophages immune cell response

## Abstract

*PIK3CA*-related overgrowth spectrum (PROS) comprises a group of rare genetic disorders caused by de novo, mosaic, postzygotic gain-of-function mutations in the *PIK3CA* gene. These mutations arise during embryogenesis, affect multiple organ systems, and result in heterogeneous clinical phenotypes. We previously showed that primary cells derived from a PROS mouse model exhibit a metabolic shift toward aerobic glycolysis (a Warburg-like effect), accompanied by altered secretion of metabolites. Here, we observed that this metabolic reprogramming was associated with the up-regulation of key transcription factors, including cellular myelocytomatosis and hypoxia-inducible factor 1 alpha. PIK3CA hyperactivation induces a distinct microenvironment marked by metabolic dysregulation, extracellular matrix remodeling, increased cellular proliferation, elevated phosphorylated form of the histone variant H2AX levels, and enhanced macrophage infiltration, hallmarks commonly associated with increased lactate production. To further examine immune infiltration dynamics, we used a mouse model expressing a constitutively active *PIK3CA* mutation selectively in adipose tissue (Adipo^CreER^), a tissue frequently affected in PROS. Single-cell transcriptomics and flow cytometry profiling revealed that macrophages adopt an immunomodulatory phenotype, with increased infiltration of TREM2^+^ and Lyve1^hi^MHCII^lo^ macrophages, along with myeloid-derived suppressor cells, and a concurrent reduction in T-cell populations. These immune alterations parallel those observed in tumor microenvironments and may contribute to tissue overgrowth and impaired immune surveillance. Multiplex immunofluorescence analysis of tissue samples from individuals with PROS confirmed these findings, underscoring the translational relevance of the mouse model. Together, our results demonstrate that *PIK3CA* mutations in PROS profoundly remodel the tissue microenvironment and reprogram macrophage function in a manner reminiscent of tumor biology.

Significance statement *PIK3CA*-related overgrowth spectrum (PROS) is caused by activating mutations in PIK3CA that drive abnormal tissue growth, yet the mechanisms linking altered metabolism to immune dysfunction remain poorly understood. We show that PIK3CA hyperactivation induces a Warburg-like metabolic program that profoundly remodels the tissue microenvironment. This metabolic reprogramming promotes extracellular matrix changes, DNA damage, and immune infiltration characterized by immunomodulatory macrophages and myeloid-derived suppressor cells, alongside reduced T-cell populations. These features closely resemble those observed in tumor microenvironments. Using single-cell analyses in mouse models and validation in human PROS tissues, our findings reveal that metabolic dysregulation in PROS reshapes local immunity and tissue homeostasis. This work establishes conceptual parallels between PROS and cancer biology and identifies immune-metabolic pathways as potential therapeutic targets.

## Introduction

*PIK3CA* encodes a ubiquitously expressed lipid kinase that regulates fundamental signaling pathways controlling cell growth, proliferation, motility, survival, and metabolism (1). The gene encodes the 110 kDa catalytic subunit of phosphoinositide 3-kinase (PI3K), p110α. Upon activation, primarily by receptor tyrosine kinases, this plasma membrane–localized enzyme catalyzes the conversion of phosphatidylinositol 4,5-bisphosphate (PtdIns(4,5)P_2_) to phosphatidylinositol 3,4,5-trisphosphate (PtdIns(3,4,5)P_3_ or PIP_3_). PIP_3_ recruits phosphoinositide-dependent kinase-1, which phosphorylates and activates AKT at Thr308, ultimately promoting mTOR activation (1).

Given its essential role in cell physiology, p110α is one of the most frequently mutated oncogenic proteins (2). Activating mutations in PIK3CA are common in cancer, accounting for ∼39.7% of oncogenic alterations across various types of tumors (3). However, it was not until the advent of next-generation sequencing in 2012 that *PIK3CA* mutations were also linked to a spectrum of congenital disorders known as *PIK3CA*-related overgrowth spectrum (PROS) (4, 5). These syndromes are caused by somatic gain-of-function mutations arising early in development, most often during zygote formation (6–10).

Despite sharing mutational hotspots with cancer, including glutamate residues 542 and 545 in the helical domain and histidine 1047 in the kinase domain, PROS patients do not exhibit increased cancer susceptibility (11). This discrepancy may stem from key differences in the context of mutation, such as the embryonic origin of the affected tissues (mesodermal and neuroectodermal in PROS versus predominantly endodermal in cancer), the surrounding tissue environment (12), and the presence or absence of cooperating mutations (13).

Most studies of *PIK3CA* mutations, whether in cancer or PROS, have focused on their cell-autonomous effects on growth and metabolism. Less is known about non-cell-autonomous consequences, particularly their impact on the tumor immune microenvironment (TIME). TIME is a complex milieu composed of tumor cells, immune cells, blood vessels, and extracellular matrix, all of which interact to shape the immune response (14). In models of colon and breast cancer, the *PIK3CA* H1047R mutation has been shown to promote an immunosuppressive myeloid microenvironment, marked by increased infiltration of myeloid-derived suppressor cells (MDSCs) (15, 16).

We recently reported that isolated *PIK3CA* gain-of-function mutations in adipose tissue induce a Warburg-like metabolic shift, characterized by up-regulation of glycolytic enzymes and increased lactate production (17). Elevated lactate concentrations in the tumor microenvironment have been implicated in macrophage polarization toward a tolerogenic, tumor-promoting phenotype (18–20). Although the PI3Kδ and PI3Kγ isoforms are more prominently expressed in leukocytes (21), these observations collectively suggest a role for p110α in modulating TIME. This warrants further investigation.

Here, we explore the impact of *PIK3CA* mutations on the cellular microenvironment, aiming to identify novel therapeutic targets for patients with PROS.

## Methods

### Mice

R26StopFLP110* mice (stock no. 012343) were crossed with Adiponectin CreER mice (stock no. 025124) obtained from The Jackson Laboratory (16). This breeding strategy generated R26StopFLP110*+/− × Adipo CreER + mice (designated Adipo^CreER^) and Cre-negative littermates (Adipo^WT^) (15, 16). The p110* mutant used in this study produces a stronger activation of the AKT/mTOR signaling pathway than commonly observed human PIK3CA variants (15, 16). Fibroblasts used for in vitro metabolic experiments were isolated from a PIK3CA^CAGG-CreER^ mouse model.

The R26StopFLP110* allele encodes a constitutively active form of PIK3CA (p110*), consisting of the p85 iSH2 domain fused to the N-terminus of p110 through a flexible glycine linker. Tissue-specific expression is achieved through a loxP-flanked neoR STOP cassette inserted into a modified pROSA26 locus, followed by the p110* cDNA, an frt-flanked IRES-EGFP cassette, and a bovine polyadenylation sequence. To monitor Cre-mediated recombination, these mice were crossed with Gt(ROSA)26Sortm4(ACTB-tdTomato, -EGFP)Luo/J reporter mice. In this system, cells initially express membrane-localized tdTomato, which switches to GFP expression after recombination.

Animals were maintained at a constant ambient temperature under a 12-h light/dark cycle with free access to food and water. Mice were fed a standard chow diet (2918 Teklad Irradiated Global 18% Protein Rodent Diet, 3.1 kcal/g; Envigo). All procedures were approved by the French Ministry of Higher Education, Research, and Innovation (APAFIS no. 27498-2020090117341716v2) and conducted in accordance with animal welfare regulations.

For in vivo experiments, 6-week-old Adipo^WT^ and Adipo^CreER^ mice received tamoxifen at a dose of 40 mg/kg per day for 5 consecutive days by oral gavage.

Mice were fasted for 12 h before sacrifice, and blood glucose was measured with an Accu-Chek Performa device (Roche Diagnostics).

### Lactate concentration

To calculate lactate concentration, we used L-Lactate Assay Kit (Colorimetric) AB65331 from Abcam following manufacture’s protocol. Lactate was calculated for Adipo^CreER^ mice plasma and primary fibroblasts derived from PIK3CA^WT^ and PIK3CA^CAGG-CreER^ mice after being induced and kept for 48 h in low-glucose media.

### Meso Scale Diagnostics U-PLEX assay

Levels of interferon-gamma (INF-γ), interleukin (IL)-1β, IL-2, IL-4, IL-6, IL-10, IL-12p70, IL-13, IL-21, and tumor necrosis factor-alpha (TNF-α; Meso Scale Diagnostics [MSD], K15069M-1) were measured for *Adipo^WT^* and *Adipo^CreER^* mice plasma which was collected at the end of each in vivo experiment in heparin-coated tubes following the manufacture’s protocol. IL-2, IL-4, IL-12p70, IL-13, and IL-21 plasma concentrations fell below the threshold level.

### Immunohistochemistry and immunofluorescence

Paraffin-embedded sections (4 μm thick) underwent antigen retrieval by heating at 120 °C in citrate buffer using a pressure cooker. For immunofluorescence, samples were permeabilized with 0.3% Triton X-100 for 15 min, followed by three washes in phosphate-buffered saline (PBS). Sections were then blocked for 1 h at room temperature in a solution containing 5% fetal bovine serum (FBS) and 1% bovine serum albumin (BSA) in tris-buffered saline (TBS). Primary antibodies (listed in Table S1) were applied overnight at 4 °C. The following day, Alexa Fluor–conjugated secondary antibodies (Thermo Fisher Scientific) were incubated with the samples. This staining procedure was applied to both tissue sections and cultured cells.

Fluorescence images were acquired using either an LSM 700 confocal microscope (Zeiss) or a Zeiss spinning disk system for mitochondrial imaging. For immunohistochemistry, detection was carried out using horseradish peroxidase–conjugated secondary antibodies with 3,3′-diaminobenzidin (DAB) as the chromogen, and images were captured using an E800 microscope (Nikon).

For each immunofluorescence staining, nuclear intensity was measured using a specific script on QuPath version 0.4.2, and cytoplasmatic intensity was measured using Image J v1.53.

For mitochondria structural evaluation, we used tetramethylrhodamine ethyl ester (TMRE) (abcam ab113852) for membrane potential and MitoTracker (Invitrogen M46753) for mitochondrial mass evaluation. The Mitochondrial Network Analysis (MiNA) plugin for ImageJ (22) v3.0 was used to quantify mitochondrial mass after spinning disk acquisition.

### mRNA analysis

Total RNA was isolated from mouse tissues and cultured cells using the NucleoSpin RNA kit (Macherey-Nagel, MANA740955), according to the manufacturer's protocol. Gene expression levels were then quantified by reverse transcription followed by quantitative PCR (RT-qPCR) using a CFX Connect system (Bio-Rad). Primer sequences used for amplification are provided in Table S2.

### Western blot

Protein extracts from primary fibroblasts and tissues of PIK3CA^WT^ and PIK3CA^CAGG-CreER^ mice were used for western blot analysis. Tissues were first mechanically homogenized and then lysed in radio-immunoprecipitation assay buffer (RIPA) buffer supplemented with protease and phosphatase inhibitors. Protein concentrations were measured using a bicinchoninic acid assay (Pierce). Equal amounts of protein were separated by sodium dodecyl sulfate–polyacrylamide gel electrophoresis and transferred to membranes.

Membranes were incubated with the appropriate primary antibodies (listed in Table S1), followed by horseradish peroxidase–linked secondary antibodies (1:10,000). Signal detection was performed using a ChemiDoc MP Imaging System, and band intensities were quantified with Image Lab software (Bio-Rad Laboratories).

### Isolation of adipose tissue-derived leukocytes and flow cytometry

Epididymal white adipose tissue (WAT) was collected from mice euthanized by cervical dislocation, excising the depot located adjacent to the epididymis. The tissue was transferred to a 1-mL tube, finely minced with scissors, and enzymatically digested in RPMI 1640 containing 10% FBS, 0.2 mg/mL collagenase type IV (Sigma, C5138), and 0.05 mg/mL DNase I (Roche, 10104159001) for 60 min at 37 °C. Following digestion, samples were passed through a 100-μm cell strainer and centrifuged at 400 × *g* for 4 min, with the temperature decreasing from room temperature to 4 °C during the process.

Cell pellets were resuspended in 500 μL of red blood cell lysis buffer (Sigma) and incubated on ice for 2–5 min, followed by washing. The resulting single-cell suspension was then incubated with a panel of specific antibodies for 30 min at 4 °C. Data acquisition was performed using a Cytek Spectral Cell Analyzer, and analysis was conducted with FlowJo software (v10.10.0).

For flow cytometry of primary fibroblasts, cells were mechanically detached, resuspended in PBS as a single-cell suspension, and stained with the appropriate antibody cocktail in fluorescence-activated cell sorting (FACS) buffer (PBS containing 0.5–1% BSA). Samples were acquired using a Sony SP6800 Spectral Cell Analyzer and analyzed with Kaluza software (v2.1). Antibodies used in this study are listed in Table S3.

### Reactive oxygen species quantification

To quantify reactive oxygen species (ROS) accumulation inside primary cells, we used CellRox Deep Red oxidative stress reagent (1 μM; Invitrogen C10422) following the manufacturer's protocol.

### Seahorse experiment

Metabolic flux analysis was performed on fibroblasts derived from PIK3CA^WT^ and PIK3CA^CAGG-CreER^ mice using a Seahorse XFe96 extracellular flux analyzer (Agilent Technologies, USA). This platform enables real-time assessment of cellular bioenergetics by simultaneously measuring glycolytic activity and mitochondrial respiration. Oxygen consumption rate (OCR) was used as an indicator of oxidative phosphorylation, while extracellular acidification rate (ECAR) reflected glycolytic activity.

Cells were plated at a density of 25,000 cells per well in Seahorse XF96 V3 PS microplates (Agilent Technologies, 101085-004) and maintained for 48 h in low-glucose medium. One hour prior to the assay, cells were washed and incubated in bicarbonate-free XF Base Medium supplemented with 4 mM L-glutamine, 500 μM glucose, and 100 μM pyruvate for OCR measurements, or with 4 mM L-glutamine and 100 μM pyruvate only for ECAR measurements. Incubation was carried out at 37 °C in a CO_2_-free environment.

OCR was recorded under basal conditions and following sequential injections of 1 μM oligomycin (ATP synthase inhibitor), 1 μM carbonyl cyanide-p-trifluoromethoxyphenylhydrazone (FCCP) (uncoupler of oxidative phosphorylation), and a combination of 1 μM rotenone and 1 μM antimycin A (inhibitors of complexes I and III of the electron transport chain). ECAR was measured at baseline and after the addition of 10 mM glucose, 1 μM oligomycin, and 50 mM 2-deoxyglucose (2-DG), a glycolysis inhibitor.

### Targeted liquid chromatography–mass spectrometry metabolite analyses

Primary fibroblasts and corresponding culture supernatants were rapidly frozen in liquid nitrogen. Metabolites for liquid chromatography–mass spectrometry (LC–MS) analysis were extracted using a solvent mixture composed of 50% methanol, 30% acetonitrile, and 20% water. Extraction volumes were scaled according to sample size, using 1 mL per 1 × 10^6^ cells or 200 μL per 10 μL of plasma or serum. Samples were vortexed for 5 min at 4 °C, followed by centrifugation at 16,000 × *g* for 15 min at 4 °C. The resulting supernatants were collected and stored at −80 °C until analysis.

Metabolomic profiling was performed on a Q Exactive Plus Orbitrap mass spectrometer equipped with an Ion Max source and HESI II probe, coupled to a Dionex UltiMate 3000 UPLC system (Thermo Fisher Scientific). External calibration was conducted weekly using the manufacturer's standard calibration solution. A volume of 5 μL per sample was injected onto a ZIC-pHILIC column (150 × 2.1 mm, 5 μm particle size) fitted with a guard column (20 × 2.1 mm, 5 μm; Millipore). The mobile phase consisted of buffer A (20 mM ammonium carbonate with 0.1% ammonium hydroxide, pH 9.2) and buffer B (acetonitrile).

Chromatographic separation was carried out at a flow rate of 0.2 μL/min with the following gradient: 80 to 20% buffer B over 20 min, 20 to 80% buffer B from 20 to 20.5 min, followed by re-equilibration at 80% buffer B until 28 min. The mass spectrometer operated in full-scan mode with polarity switching, using a spray voltage of 2.5 kV and a capillary temperature of 320 °C. Gas settings were 20 units for sheath gas, 5 units for auxiliary gas, and 0 for sweep gas. Data were acquired across a mass range of *m*/*z* 75–1,000 at a resolution of 35,000 (at *m*/*z* 200), with an automatic gain control target of 10^6^ and a maximum injection time of 250 ms. Lock mass calibration ensured mass accuracy within 5 ppm.

Data acquisition was performed using Xcalibur software (version 4.0.27.13), and metabolite peak areas were quantified with TraceFinder (version 3.3 SP1). Metabolite identification relied on accurate mass measurements and established retention times. Pathway enrichment analysis was conducted using the MetaboAnalyst web platform (version 6.0).

### Bone marrow–derived macrophage extraction

Bone marrow (BM) cells were isolated from 10- to 16-week-old C57BL/6 mice following euthanasia. Femurs and tibias were flushed with cold PBS supplemented with 2% FBS, and the resulting cell suspension was passed through a 70-μm cell strainer. Cells were pelleted by centrifugation at 252 × *g* for 7 min at 4 °C and subjected to red blood cell lysis using red blood cell (RBC) lysis buffer (BioLegend). After washing, cells were plated in six-well plates at a density of 4 × 10^5^ cells/mL in Dulbecco's Modified Eagle Medium (DMEM) (4.5 g/L glucose) supplemented with 10% FBS, penicillin (100 U/mL), streptomycin (0.1 mg/mL), and 25 ng/mL recombinant mouse macrophage colony-stimulating factor (M-CSF) (Miltenyi Biotec, 130-101-706). Cells were cultured for 7 days to allow differentiation into macrophages.

Following differentiation, cells were maintained in medium containing 25 ng/mL M-CSF and 50 ng/mL granulocyte-macrophage colony-stimulating factor (GM-CSF) (Miltenyi Biotec, 130-095-746) in a resting state prior to experimental stimulation.

### Co-culture experiment

To create a 3D spheroid structure, primary fibroblasts (PIK3CA^WT^ and PIK3CA^CAGG-CreER^ mice derived) were collected and re-suspended at the concentration of 8 × 10^5^ cells/mL in complete DMEM 4.5 g/L 10% FBS, P/S media supplemented with tamoxifen to induce PIK3CA overactivation. They were seeded in 25 µL drops upside down on the lid of a 100-mm petri dish (hanging drop culture (23)). After 5 days spheroids were collected and resuspended in 100 µL of matrigel (Corning, 356234). Matrigel-embedded spheroids were plated in a 12-well plate and kept in the incubator for 30 min before adding 1 mL of 8 × 10^5^ cell/mL BM-derived macrophages. Fibroblasts and macrophages were kept in co-culture for 6 days before proceeding with flow cytometry analysis.

### Cell migration experiment

A scratch assay was used to quantify macrophage migration in vitro. Briefly, BM-derived macrophages were grown until reaching confluence. Using a 200-µL tip, an individual scratch was performed in each well. Cells were washed with PBS, and a picture was taken to mark time 0. PBS was then substituted with conditioned media derived from primary fibroblasts treated for 24 h with 10 µM of Stiripentol (MedChemExpress, cat. no.: HY-103392) or left untreated, followed by a 24-h starvation. Images were taken again after 24 and 48 h. Quantifications of the wounded area were performed using ImageJ/Fiji plugin “Wound_healing_size_tool” (24).

### Single-cell RNA sequencing and library preparation

White adipose tissue was enzymatically digested as described for flow cytometry preparation. Prior to downstream analysis, CD45^+^ cells were enriched using CD45 microbeads (Miltenyi Biotec) in combination with an AutoMACS separator. Single-cell transcriptomic profiling was carried out using the BD Rhapsody platform, following the manufacturer's instructions (BD Biosciences).

A total of 46,500 viable cells were captured in a single run, with two barcoded samples pooled together (23,250 cells per condition). Each condition consisted of cells pooled from three mice. Library preparation was performed using the BD Rhapsody Whole-Transcriptomic Analysis Amplification Kit (633801) and Reagent Kit (665915).

Sequencing of mRNA libraries was conducted using paired-end reads (2 × 151 cycles) on an Illumina HiSeq 4000 system, including a 20% PhiX control spike-in. Quality control assessments were performed at each step of the workflow.

### Processing and analysis of transcriptomic data

All computational analyses were conducted using R (version 4.4.1). Sequencing reads were aligned to the mouse reference genome (GRCm38_M13, GENCODE) using STAR (version 2.5.3a) (25), and transcript abundance was quantified as transcripts per million with RSEM (26). Downstream single-cell analyses, including dimensionality reduction (principal component analysis [PCA], t-distributed stochastic neighbor embedding [t-SNE], and uniform manifold approximation and projection [UMAP]), clustering, heatmap generation, cell population proportions, and identification of differentially expressed genes, were performed using Seurat (version 5.1.0) (27).

Optimal clustering resolution was determined using the clustree package (version 0.5.0), selecting the most stable parameter (watall_snn_res0.3). Differential gene expression was assessed using the Wilcoxon rank-sum test implemented in Seurat via the “FindAllMarkers” function. Initial cell-type annotation was carried out manually based on reference datasets available through the Broad Institute Single Cell Portal, and assignments were further validated through comparison with previously published datasets (28–30). Visualization of gene expression patterns was performed using violin plots and UMAP feature plots generated with the “VlnPlot” and “FeaturePlot” functions.

Gene set enrichment analysis for each identified cluster was conducted using the Metascape web platform (version 3.5) (31).

### Primary cell cultures

Primary fibroblasts were isolated from PIK3CA^WT^ and PIK3CA^CAGG-CreER^ mice, as previously established (7). Briefly, skin biopsies were collected, finely minced, and incubated for 30 min at room temperature in 0.05% trypsin-ethylenediaminetetraacetic acid (EDTA) (Thermo Fisher Scientific) with gentle agitation. Following centrifugation at 700 × *g* for 10 min, cells were resuspended in high-glucose DMEM (4.5 g/L; Corning) supplemented with penicillin (100 IU/mL), streptomycin (500 μg/mL; Gibco), and 20% FBS (Sigma) and plated in T75 flasks to establish primary cultures.

Cells were subsequently maintained in DMEM containing 10% FBS and 1× penicillin/streptomycin. Experiments were performed using cells between passages 2 and 5. Unless otherwise specified, cells were cultured in low-glucose conditions using glucose-free DMEM (Gibco) supplemented with 10% FBS, 50 μM pyruvate, and 1× penicillin/streptomycin. To induce Cre recombination, culture medium was replaced with fresh medium containing 1 μM 4-hydroxytamoxifen (Sigma-Aldrich) for 48 h prior to experimentation.

For western blot and immunofluorescence analyses, cells were serum-starved in DMEM without FBS for 6–12 h, followed by treatment for 24 h with either BYL719 (1 μM; MedChem Express) or an equivalent volume of dimethyl sulfoxide (DMSO) as control. Cells were then washed with PBS, and protein extracts were obtained by direct lysis in RIPA buffer supplemented with protease and phosphatase inhibitors. For inhibition of GSK3β, cells were treated with Laduviglusib (3 μM) for 3 h prior to protein extraction. Each experiment was conducted in duplicate and independently repeated at least three times.

### BrdU staining

Mice were injected 24 h before euthanizing with 100 mg/kg of BrdU (Invitrogen B23151) and starved overnight. Extracted tissue was paraffin embedded and cut for BrdU staining following the classical immunofluorescence protocol that we described above.

### Patients

Paraffin-embedded human tissue sections were dewaxed, subjected to antigen retrieval, and blocked prior to staining. Multiplex immunofluorescence was performed using the PhenoCode Signature panel for human M1/M2 polarization (AKOYA Biosciences, six-plex panel targeting CD8, CD68, and CD163; cat# PCSPP004), following the manufacturer's instructions.

In this approach, antibodies conjugated to distinct oligonucleotide barcodes were applied simultaneously in a single incubation step. Signal detection was carried out sequentially, with each antibody revealed individually through horseradish peroxidase–mediated amplification using Opal fluorophores. This cycle was repeated until all markers were visualized on the same tissue section. Details of the antibodies and their working concentrations are provided in Table S4.

Control samples consisted of nondiseased tissues obtained during routine genetic diagnostic procedures. The study protocol was approved by the Necker Hospital Institutional Review Board (approval number 22.01638.000089). Samples de-identified prior to use.

### Statistical analyses

Statistical analyses were conducted using GraphPad Prism (version 10.0). Data are presented as mean ± SEM. Comparisons among multiple groups were performed using ANOVA followed by Tukey–Kramer post hoc testing when appropriate. For comparisons between two groups, either unpaired *t*-tests or Mann–Whitney tests were applied depending on data distribution. Differences were considered statistically significant at *P* < 0.05.

## Results

### PIK3CA overactivating mutation drives metabolic changes toward a Warburg-like effect

**Figure 1:**
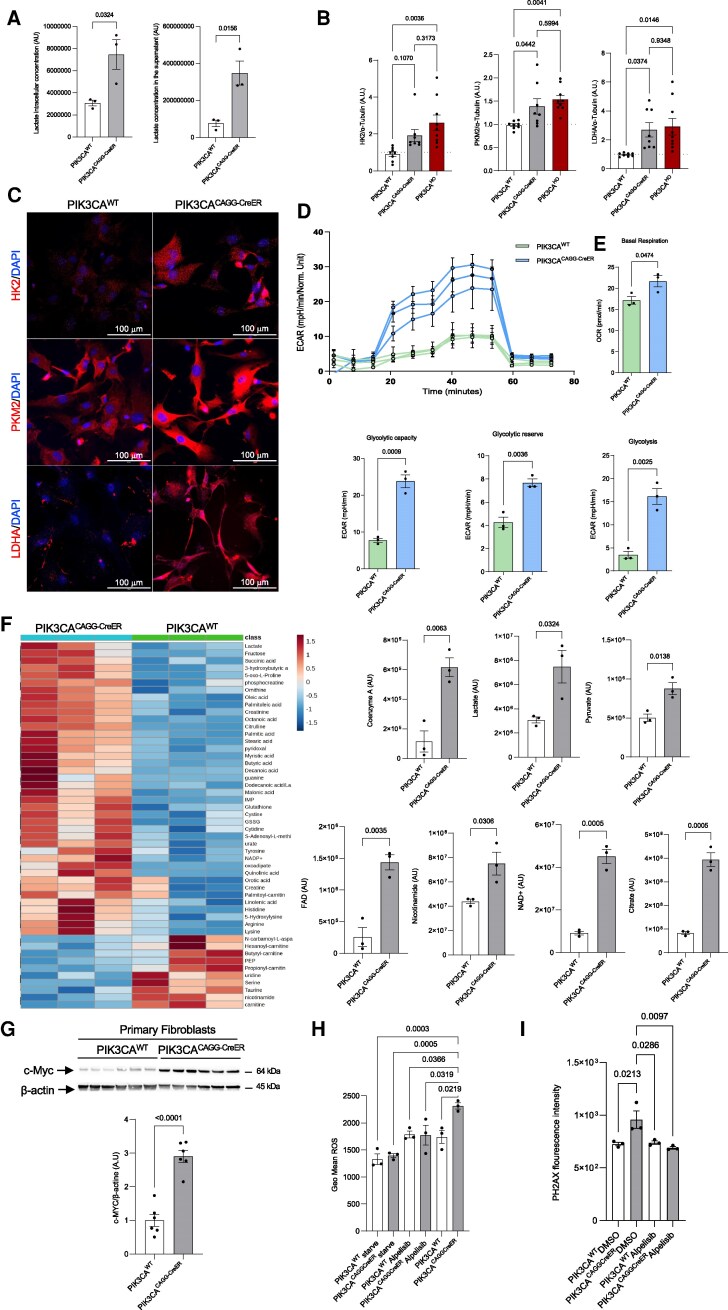
PIK3CA activating mutation induces a metabolic shift toward a Warburg-like effect. A) Lactate concentrations in PIK3CA^WT^ and PIK3CA^CAGG-CreER^ primary fibroblast (left panel) corresponding culture supernatants under low-glucose conditions (right panel) (*n* = 3 per group). B) Western blot quantification of HK2, PKM2, and LDHA in primary fibroblasts kept in low-glucose conditions derived from PIK3CA^WT^, PIK3CA^CAGG-CreER^, and PIK3CA^HO^ mice (*n* = 8 to 9 per group). C) Representative immunofluorescence images showing HK2, PKM2, and LDHA expression in primary fibroblasts from PIK3CA^WT^ and PIK3CA^CAGG-CreER^ mice cultured under low-glucose conditions. D) Basal levels of ECAR were measured using an extracellular flux analyzer (Seahorse Bioscience) in PIK3CA^WT^- and PIK3CA^CAGG-CreER^-derived primary fibroblasts. Glycolysis was calculated after the addition of 10 mM of glucose; maximal ECAR, that is, glycolytic capacity, was stimulated by the addition of 1 µM oligomycin A, glycolytic reserve was induced by the addition of 2-DG 50 mM. E) The basal mitochondrial OCR of primary fibroblasts was derived by subtracting nonmitochondrial OCR (remaining OCR after antimycin A addition). Data from one replicative experiment are shown (*n* = 3 per condition). F) Heatmap of the 50 top metabolite changes observed in PIK3CA^WT^ and PIK3CA^CAGG-CreER^ fibroblasts (*n* = 3 per condition) cultured in low-glucose conditions. On the right are reported the graphics in arbitrary units (AU) of some of the most relevant metabolites. G) Western blot and quantification of c-Myc in primary fibroblasts in low-glucose conditions derived from PIK3CA^WT^ and PIK3CA^CAGG-CreER^ mice (*n* = 6 per group). H) Flow cytometry analysis of ROS content inside primary fibroblasts in low-glucose conditions of PIK3CA^WT^ and PIK3CA^CAGG-CreER^ mice (*n* = 3 per group). Fibroblasts were either starved for 24 h, treated with 1 µM alpelisib for 24 h, or left untreated. I) Quantification of PH2AX fluorescence intensity of primary fibroblasts in low-glucose conditions of PIK3CA^WT^ and PIK3CA^CAGG-CreER^ mice treated with DMSO or alpelisib 1 µM for 24 h as calculated by QuPath algorithm, 10 images for sample (*n* = 3 per group).

We recently reported that patients with PROS involving adipose tissue display hormonal dysregulation and metabolic reprogramming consistent with a Warburg-like effect (17). To investigate the cellular mechanisms underlying lactate production, we isolated primary fibroblasts from a PROS mouse model (hereafter referred to as PIK3CA^CAGG-CreER^) (7) and cultured them under low-glucose conditions (<1 mM), reflecting the in vivo environment observed in both humans and mice with the disease (16). Fibroblasts from PIK3CA^CAGG-CreER^ mice showed increased lactate production compared with wild-type controls (Fig. 1A). This was accompanied by elevated expression of glycolytic enzymes hexokinase 2 (HK2), pyruvate kinase muscle isoform 2 (PKM2), and lactate dehydrogenase A (LDHA) (Figs. 1B, C and S1A, B), a phenotype further enhanced in mice homozygous for the mutation (*PIK3CA^HO^*; Figs. 1B and S1A, B). It is important to note that homozygous mutations of PIK3CA have not been reported in humans. However, in mice, homozygous mutations enhance activation of the AKT/mTOR pathway and exacerbate disease-associated phenotypes (15, 16). Seahorse metabolic flux analysis confirmed an increase in glycolytic capacity and reserve in mutant cells, indicated by elevated ECARs (Fig. 1D). These metabolic changes were not due to alterations in mitochondrial morphology or organization (Fig. S1C–E). OCR and basal respiration were significantly altered in fibroblasts derived from PIK3CA^CAGG-CreER^ mice (Figs. 1E and S1F). Metabolite profiling revealed sustained aerobic glycolysis driven by increased NAD^+^ levels, resulting in elevated pyruvate and intermediates of anabolic pathways, including the pentose phosphate pathway, alongside accumulation of amino acids (leucine, isoleucine, and tryptophan) and fatty acids (coenzyme A, carnitine, citrate, eicosapentaenoic acid, and palmitoleic acid; Fig. 1F). High levels of NAD^+^ and FAD^+^ were consistent with a need to maintain redox homeostasis. Notably, these metabolic features resemble those observed in cancer cells undergoing the Warburg effect (32–34) (Figs. 1F, S1G, H, S2, and S3).

To understand how p110α activation drives glycolysis, we examined the expression of 6-phosphofructo-2-kinase/fructose-2,6-bisphosphatase 3 (PFKFB)3, cellular myelocytomatosis (c-Myc), and hypoxia-inducible factor 1 alpha (HIF-1α), key regulators of the metabolic switch in highly glycolytic tumors. To explore these phenomena, we used fibroblasts derived from the PIK3CA^CAGG-CreER^ mouse model. We previously reported that these cells recapitulate, at the metabolic level, the alterations observed in adipocytes, exhibiting a Warburg-like effect (16). All were up-regulated in mutant fibroblasts (Figs. 1G, S1I, and S4A). c-Myc activation appeared partially mediated by AKT-dependent inhibition of glycogen synthase kinase 3 beta (GSK3β) (Fig. S4B and C). Increased metabolic flux is frequently linked to ROS accumulation. In mutant fibroblasts, metabolomic analysis revealed increased glutathione disulfide and reduced levels of reduced glutathione (Fig. S4D), indicative of oxidative stress. Flow cytometry confirmed elevated ROS levels (Fig. S4E), which were attenuated by prolonged nutrient deprivation or treatment with the PI3Kα inhibitor alpelisib (Fig. 1H).

Given the established role of ROS in DNA damage (35), we assessed the DNA damage response in PIK3CA^CAGG-CreER^ fibroblasts. Immunofluorescence revealed elevated phosphorylated form of the histone variant H2AX (γH2AX) staining relative to controls (Fig. S5A), which was also observed in liver tissue from mutant mice (Fig. S5B). Alpelisib treatment reduced both ROS and γH2AX levels (Fig. 1H and I), suggesting that PI3K activity links metabolic reprogramming to DNA damage signaling. Elevated γH2AX may also reflect replication stress in the rapidly proliferating mutant cells (Fig. S5C).

Finally, to further validate the role of PI3K in driving this phenotype, we treated mutant fibroblasts with alpelisib and observed a reduction in glycolytic enzyme expression, notably HK2 and LDHA, as shown by immunoblotting (Fig. S5D and E), consistent with previously reported decreases in lactate production (16). Together, these results demonstrate that p110α activation alone is sufficient to drive aerobic glycolysis, ROS accumulation, and DNA damage in fibroblasts through a PI3K-dependent mechanism.

### PIK3CA overactivating mutation drives in vitro and in vivo macrophage infiltration and reprogramming

**Figure 2:**
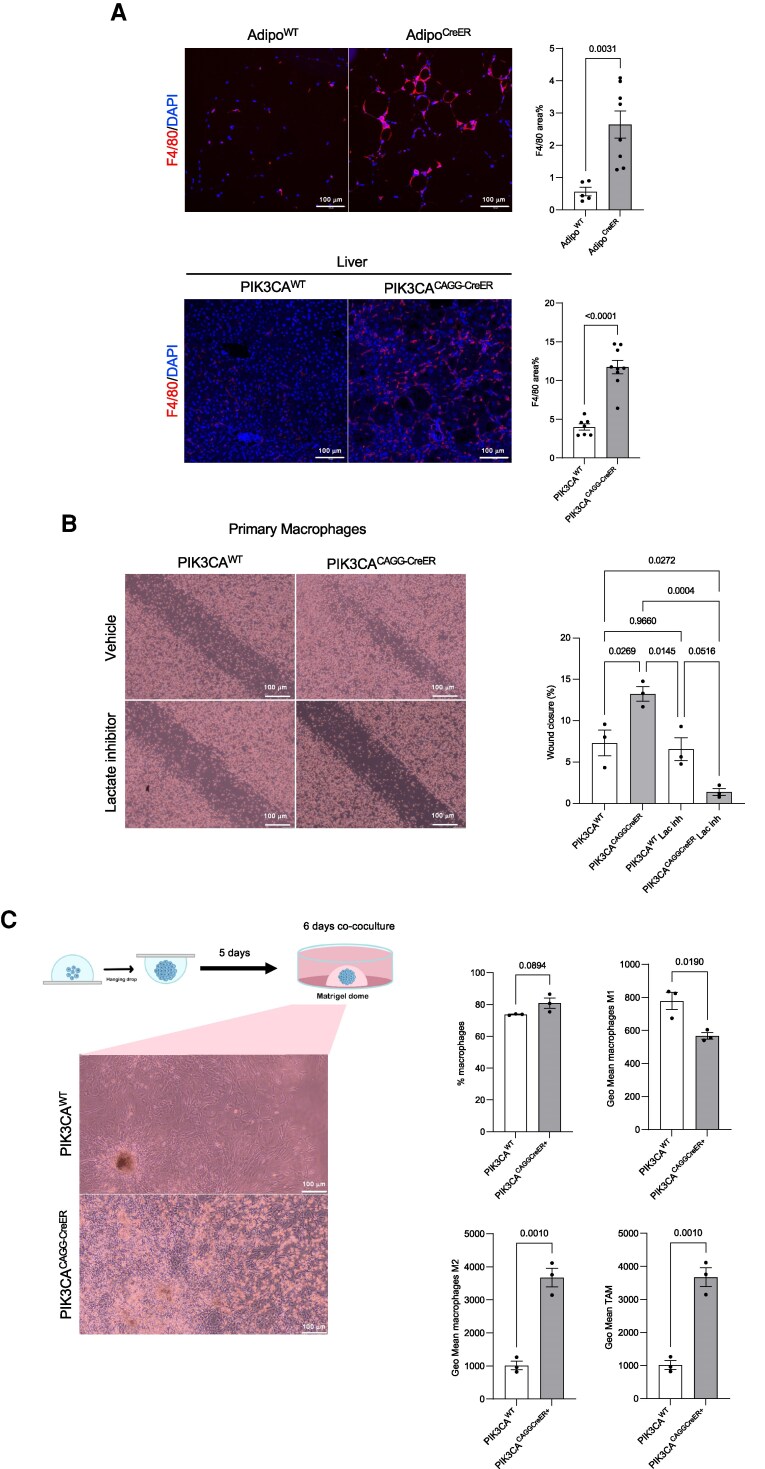
Activating PIK3CA mutation drives macrophage infiltration and reprogramming in vitro and in vivo. A) F4/80 immunofluorescence and quantification in adipose tissue from Adipo^WT^ and Adipo^CreER^ (upper panel) and in the liver of PIK3CA^CAGG-CreER^ and PIK3CA^WT^ mice (lower panel). *N* = 5 Adipo^WT^ and *n* = 8 Adipo^CreER^ mice; *n* = 7 PIK3CA^WT^ and *n* = 9 PIK3CA^CAGG-CreER^. B) Representative images and quantification of relative (48 h–*t*0) wound healing of primary macrophages from a scratch assay analysis following stimulation with CM from PIK3CA^WT^ and PIK3CA^CAGG-CreER^ primary fibroblasts treated with or without Stiripentol (10 mM; *n* = 3 samples per group). C) Experimental design of the coculture experiments, representative images, and macrophage phenotyping assessed by flow cytometry (*n* = 3 samples per group). TAM, tumor-associated macrophage.

Since we observed that *PIK3CA* mutation in the context of PROS leads to changes in the metabolism of the affected cells, notably characterized by an increase in lactate production both inside and outside of the cell (Fig. 1A), we decided to explore more in detail how this influences the cellular microenvironment. We previously reported a dramatic increase in macrophage infiltration in the adipose tissue and liver in two published mouse models carrying a *PIK3CA* mutation: one with the mutation in adipose tissue (*Adipo^CreER^*) and the other in mosaic PROS (PIK3CA^CAGG-CreER^) (7, 16). We first confirmed using immunofluorescence and flow cytometry experiments the increased infiltration of macrophages in the affected tissues of both mouse models compared with controls (Figs. 2A, S6A, B, and S7A). To better characterize this infiltration and its relationship with the PIK3CA mutation, we investigated whether any of the most enriched metabolites identified in our metabolomics experiment might also play a role in immune cell chemoattraction and polarization. In cancer and other contexts (18–20, 36, 37), aerobic glycolysis and lactate production promote macrophage infiltration and contribute to their polarization toward an immunosuppressive phenotype. Interestingly, lactate emerged as one of the most enriched metabolites in *PIK3CA*-mutated fibroblasts (Fig. S1H). For this reason, we decided to investigate its role as a chemoattractant in our models. To investigate this, we isolated BM-derived macrophages from C57BL/6 wild-type mice and differentiated them into unstimulated macrophages using M-CSF and GM-CSF for 7 days. To assess whether *PIK3CA*-mutant fibroblasts exhibit enhanced chemoattractant capacity due to increased lactate production, we treated the macrophages for 48 h with conditioned media (CM) from PIK3CA^CAGG-CreER^ or PIK3CA^WT^ primary mouse fibroblasts. Macrophages exposed to CM from mutant fibroblasts showed significantly greater wound closure (Fig. 2B). Inhibition of lactate production using an LDHA inhibitor (Lac inh) markedly reduced wound closure in both conditions (Fig. 2B).

We next examined the impact of the overactive PIK3CA mutation on macrophage phenotype. Wild-type primary macrophages were co-cultured with either PIK3CA^WT^ or PIK3CA^CAGG-CreE*R*^ primary fibroblasts. Spheroid-like structures were generated using the hanging drop method to mimic in vivo conditions. After 5 days, spheroids were embedded in Matrigel and, once solidified, overlaid with unstimulated primary macrophages. Following 6 days of co-culture, mutant fibroblasts induced marked macrophage infiltration into the Matrigel dome and promoted polarization toward a tumor-associated macrophage (TAM)-like phenotype. This shift was characterized by increased CD206 expression and reduced CD11c expression, indicative of an immunosuppressive (M2-like) profile (Figs. 2C and S6B).

Together, these findings suggest that lactate production by *PIK3CA*-mutant cells promotes macrophage recruitment and drives their polarization toward an immunosuppressive phenotype.

### PIK3CA mutation recruits MDSCs, lipid-associated Trem2+ and Lyve1+ macrophages to shape the immunosuppressive tumor microenvironment

**Figure 3:**
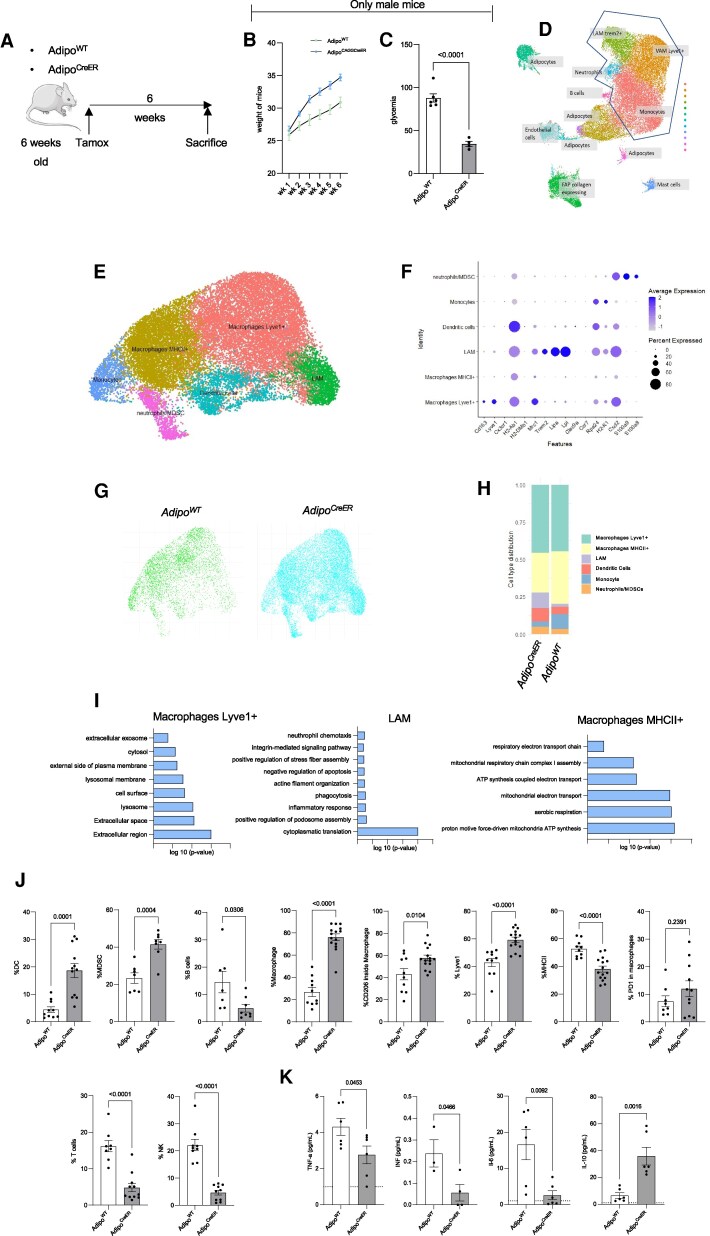
PIK3CA mutation in PROS recruits MDSCs and M2 macrophages to establish an immunosuppressive microenvironment. A) Experimental design: 6-week-old Adipo^WT^ and Adipo^CreER^ mice were treated with tamoxifen to induce the mutation and sacrificed 6 weeks later. B) Weight gain of Adipo^WT^ (*n* = 6 males) and Adipo^CreER^ (*n* = 4 males) mice after induction. C) Glycemia at the time of sacrifice (*n* = 6 Adipo^WT^ male, *n* = 4 Adipo^CreER^ male). D) UMAP plot and cluster annotation of CD45-enriched cells derived from Adipo^WT^ and Adipo^CreER^ mice adipose tissue (*n* = 3 per group). E) UMAP plot and cluster annotation of myeloid compartment after filtering. F) Cluster identities are based on the different expressions of well-known marker genes. G) UMAP plot and cluster annotation of myeloid compartment distinct for Adipo^WT^ (green) and Adipo^CreER^ (light blue) mice. H) Cluster distribution in Adipo^WT^ and Adipo^CreER^ mice. Cluster identities were based on the expression of key markers highlighted in (F). I) Gene ontology analysis using DAVID Gene Functional Classification Tool. J) Flow cytometry quantification of B cells, DCs, MDSCs, the different macrophage populations, T and NK cells infiltration in Adipo^WT^ (*n* = 7), and Adipo^CreER^ (*n* = 8) adipose tissue. K) TNF-α, INF, IL-6, and IL-10 measurements in serum derived from Adipo^WT^ (*n* = 3–7) and Adipo^CreER^ mice (*n* = 4–6).

To further investigate immune cell dynamics in vivo, we sought to characterize the immune infiltration associated with p110α overactivation. Although the role of the immune system in PROS progression remains poorly understood, we hypothesized that hyperactive p110α signaling may reshape the immune microenvironment. Given the frequent involvement of adipose tissue in PROS, we used male Adipo^CreER^ mice and performed single-cell RNA sequencing on WAT collected from 12-week-old Adipo^CreER^ and littermate male controls (Adipo^WT^) following tamoxifen induction (Fig. 3A). We used male mice because we had already observed that the phenotype was more pronounced in males compared with females (16).

By 2 weeks postinduction, Adipo^CreER^ mice began to exhibit increased body weight (Fig. 3B), and by 6 weeks, when the phenotype was fully established, as evidenced by hypoglycemia (Fig. 3C), adipose tissue was harvested. CD45^+^ immune cells were isolated, yielding 46,500 live cells from three biological replicates per group. After cell hashing and in silico demultiplexing, 30,447 high-quality cells were retained for downstream analysis using Seurat v4.0 (31). Principal component analysis and unsupervised clustering identified 12 immune populations, which were annotated based on canonical markers and reference datasets (Figs. 3D and S7A–C).

Focusing on myeloid infiltration, we excluded nonmyeloid clusters and reclustered the data, identifying one monocyte, three macrophage, one dendritic cell (DC), and one neutrophil population (Fig. 3E). Monocytes were marked by elevated *H2-K1*, *Rps24*, *Rpl23*, *Fn1*, *Clec4e*, and *Plac8* expression. DCs expressed *Cxcl2*, *H2-Ab1*, Xcr1, and Ccr7. Neutrophils displayed an MDSC-like phenotype, expressing *S100a8*, *S100a9*, and elevated *Cd84*, *Clec4e*, *Il1b*, and *Cxcl2*.

Among macrophages, we identified: (i) Lyve1^hi^MHCII^lo^ vascular-associated macrophages (VAMs), enriched in *Cd163*, *Lyve1*, *Cd209f*, and *Mrc1*, consistent with immunoregulatory and wound-healing roles; (ii) Lyve1^lo^MHCII^hi^ macrophages with high antigen-presenting capacity, expressing *H2-Ab1* and *H2-DMb1*, both subsets resembling tissue-resident, monocyte-derived macrophages conserved across tissues; and (iii) lipid-associated macrophages (LAMs), marked by elevated *Trem2*, *Lipa*, *Lpl*, *Lgals1*, and *Lgals3* (Fig. 3F). All three macrophage subsets were more abundant in *Adipo^CreER^* mice, consistent with increased macrophage infiltration (Figs. 3G and S7C).

Importantly, p110α activation reshaped the immune landscape, increasing the prevalence of LAMs, DCs, and MDSC-like neutrophils while reducing MHCII^+^ macrophages (Fig. 3H). Gene ontology analysis suggested that Lyve1^+^ macrophages and LAMs contribute to extracellular matrix remodeling, while LAMs may promote neutrophil recruitment. In contrast, MHCII^+^ macrophages up-regulated oxidative phosphorylation genes, possibly reflecting adaptation to glucose-limited conditions in the tissue microenvironment (Fig. 3I).

These findings were validated via spectral flow cytometry. Freshly isolated WAT from sacrificed mice confirmed enrichment of mast cells and neutrophils, with reduced B cells in *Adipo^CreER^* mice, consistent with single-cell data (Fig. 3J). Macrophages from mutant mice expressed elevated Lyve1, reduced MHCII, and increased surface CD206, a marker associated with TAMs (Fig. 3J). MDSC frequency was similarly increased (Fig. 3J), aligning with the transcriptomic profile.

Notably, VAMs, LAMs, and MDSCs are frequently linked to poor prognosis and immune suppression in cancer, suggesting that PIK3CA hyperactivation alone can recapitulate features of an immunosuppressive, tumor-like microenvironment. Concordantly, Adipo^CreER^ mice showed reduced infiltration of T cells and natural killer (NK) cells (Fig. 3J), a pattern also observed in tumor contexts.

Next, we examined whether the observed alterations in immune cell composition were accompanied by changes in cytokine and chemokine expression. Notably, levels of pro-inflammatory cytokines, including IFN-γ, TNF-α, and IL-6, were significantly reduced in *Adipo^CreER^* mice compared with controls (Figs. 3K and S7D).

Finally, we observed that PIK3CA-mutant adipose tissue undergoes extensive extracellular matrix (ECM) remodeling, characterized by increased fibrosis, collagen deposition, and up-regulation of select matrix metalloproteinases (MMPs; Fig. S8A–C). These changes may contribute to the formation of a fibrotic, immune-tolerant niche that promotes the persistence of mutant cells.

All together, these findings support the conclusion that p110α hyperactivation alone is sufficient to reprogram the immune milieu, promoting the establishment of an immunosuppressive and immunotolerant microenvironment reminiscent of tumor-associated immune landscapes.

### PIK3CA overactivating mutation in PROS patients induces an immunosuppressive microenvironment

**Figure 4:**
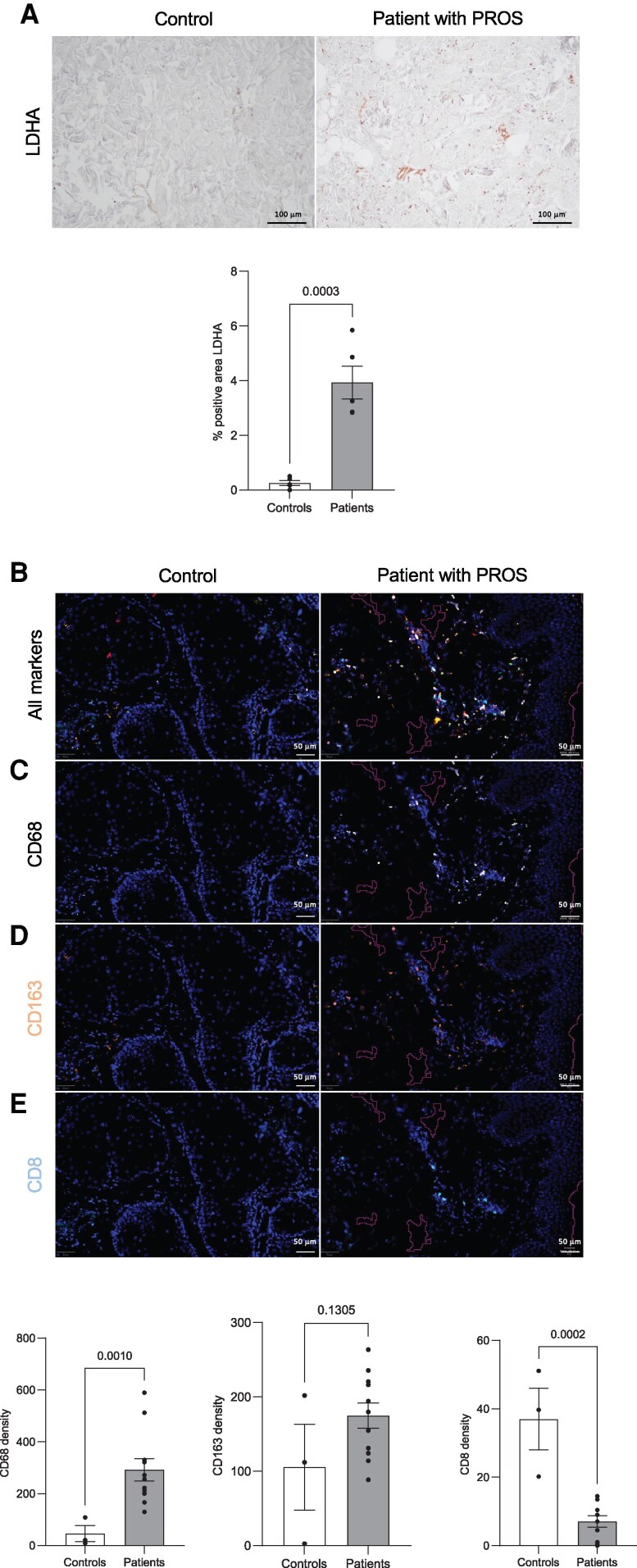
Immune modulation is also observed in tissues from patients with PROS. A) Representative LDHA immunohistochemistry of human skin biopsies from controls and patients with PROS and quantification using Colour Deconvolution 2 ImageJ plugin for DAB staining. B) Representative multiplex immunofluorescence of skin biopsies of healthy controls and patients with PROS. Merge of all the antibodies. DAPI (in blue), CD8 (light blue), CD68 (white), and CD163 (orange) scale bar, 50 μm. C–E) Representative images and quantification of single staining derived from the multiplex immunohistochemistry experiment expressed in terms of density, number of stained cells per mm^2^ of analyzed tissue (healthy controls *n* = 3, patients with PROS *n* = 10).

Finally, we sought to determine whether these findings translate to patients with PROS. We previously reported that six months of alpelisib treatment reduces serum lactate levels in PROS patients compared with baseline (17). Consistent with these metabolic alterations, skin biopsies from patients (Table S5) exhibited elevated LDHA expression relative to controls (Fig. 4A). To assess whether these metabolic changes were accompanied by immune modulation analogous to our mouse models, we performed multiplex immunofluorescence using the PhenoCode Signature M1/M2 polarization panel on skin biopsies from 10 PROS patients (Table S5) and three healthy controls. Mirroring the mouse data, PROS biopsies showed increased macrophage infiltration with a pronounced M2-like phenotype, as indicated by elevated densities of CD68^+^ and CD163^+^ cells (cells per mm^2^; Fig. 4B–D). Conversely, CD8^+^ T-cell infiltration was reduced, further supporting the immunosuppressive shift observed in the murine models (Fig. 4E).

These data indicate that patients with PROS exhibit immune compartment remodeling, particularly macrophage polarization toward an immunosuppressive phenotype, which is consistent with observations in *PIK3CA-*mutant mice.

## Discussion

Many of the clinical manifestations of PROS have been linked to cell-autonomous effects of PIK3CA mutations, such as increased cellular proliferation, hypertrophy, and tissue overgrowth. However, advances in genetic sequencing have suggested that PIK3CA mutations and PROS may also involve non-cell-autonomous mechanisms (38). In affected tissues, the mutation burden is often lower than the 50% expected if all cells were heterozygous for the mutation, and the overgrown regions typically comprise multiple cell types of distinct embryonic origins. These observations raise the intriguing possibility that PIK3CA-mutant cells may exert growth-promoting influences on neighboring or even distant wild-type cells (11). More recently, this phenomenon has been observed across various tissue types (39, 40). However, our findings reveal a more complex interplay between cell-intrinsic and cell-extrinsic mechanisms that collectively contribute to the establishment of a pro-tumorigenic, immunosuppressive microenvironment. This niche facilitates the persistence of mutated cells and shields affected tissues from immune surveillance. A central finding of our study is the metabolic reprogramming of *PIK3CA*-mutant fibroblasts and adipocytes, which adopt a Warburg-like phenotype characterized by increased aerobic glycolysis. This shift is driven by PI3K/AKT-mediated up-regulation of HIF-1 and c-Myc, key regulators of glycolytic metabolism and tumor progression. Notably, elevated lactate production, a hallmark of this metabolic state, modulates the immune microenvironment by recruiting myeloid cells and promoting their polarization toward an immunosuppressive phenotype. This mirrors similar immunometabolic interactions observed in cancer, where lactate accumulation is known to support immune evasion and tumor growth.

We also observed increased levels of γH2AX, indicative of an activated DNA damage response. This may result from accelerated G1-to-S–phase progression in mutant cells, necessitating enhanced replication stress management. In parallel, we detected up-regulation of PFKFB3, a glycolytic enzyme frequently elevated in tumors and rapidly proliferating cells, where it supports both metabolic flux and anti-apoptotic signaling.

In vivo, PIK3CA-mutant adipose tissue exhibited ECM remodeling, including increased fibrosis, collagen deposition, and up-regulation of select MMPs (7, 16). These changes contribute to the formation of a fibrotic, immune-tolerant microenvironment that may further support the survival of mutant cells. This ECM remodeling closely resembles that seen in tumor microenvironments, suggesting that PROS tissues can acquire tumor-like features without undergoing malignant transformation. Recent findings have identified a specialized subset of lymphatic endothelial cells with pronounced immune-interacting properties that shape local immune tolerance and tissue remodeling (41). The emergence or activation of such lymphatic endothelial subtypes in PIK3CA-mutant tissues could further reinforce the establishment of an immune-privileged, fibrotic niche by modulating leukocyte trafficking and antigen presentation dynamics. Perhaps most notably, despite harboring canonical oncogenic hotspot mutations, PROS patients do not exhibit an increased incidence of malignancy. This challenges conventional models of oncogenesis and highlights the critical role of tissue context, including germ layer origin (mesodermal and neuroectodermal in PROS versus endodermal in most cancers), metabolic state, and immune composition, in determining the oncogenic potential of *PIK3CA* mutations. Our data suggest that the immune landscapes of PROS and cancer are surprisingly parallel, with both skewed toward tolerance rather than immune-mediated clearance of aberrant cells. In this context, immunomodulatory therapies warrant investigation as potential treatment strategies for PROS.

Among the most promising avenues is targeting lactate metabolism. Our findings identify lactate as a key modulator of immune cell function in *PIK3CA*-mutant tissues. Inhibition of lactate production, for example, via LDHA inhibitors, may rebalance immune infiltration and activation, rendering the microenvironment less permissive to immune escape. Recent studies in glioblastoma models have shown that repurposing lactate-targeting agents such as stiripentol reduces myeloid cell infiltration and improves survival. Similar strategies could hold therapeutic potential in PROS, where dampening lactate production may enhance immune clearance of mutant cells.

In summary, our study provides new insights into the pathophysiology of PROS, demonstrating that *PIK3CA* mutations orchestrate both cell-autonomous and non-cell-autonomous changes that remodel the metabolic and immune microenvironments to favor mutation persistence. These findings open new therapeutic avenues that target the metabolic and immunological vulnerabilities of PIK3CA-mutant tissues, with the potential to improve clinical outcomes for patients with PROS.

## Supplementary Material

pgag163_Supplementary_Data

## Data Availability

All data supporting the findings of this study are available in the manuscript and on a repository site (https://doi.org/10.5281/zenodo.20070130).
