# Supplementary material for: Oncogenic PIK3CA mutations shape an immunoregulatory microenvironment in mosaic overgrowth disorders
Source: PNAS Nexus. 2026 May 13;5(6):pgag163. doi: 10.1093/pnasnexus/pgag163 (PMC13222027; doi:10.1093/pnasnexus/pgag163)
Supplement: pgag163_Supplementary_Data [file pgag163_supplementary_data.zip › PNASNEXUS-PNASNEXUS-2025-01923-TR-s02.pdf]

Supplementary Table 1: Primary and secondary antibodies used for Western blot and immunostaining in mouse experiments

Antibody target	Catalog Number	Supplier	Dilution	Application
p110α	4249	Cell Signaling Technology	1:1000	WB
P-AKT (Thr308)	13038	Cell Signaling Technology	1:1000	WB
PKM2	4053	Cell Signaling Technology	1:1000/1:100	WB/IF
P-S6RP	5364	Cell Signaling Technology	1:1000	WB
α-Tubulin	t5168	Merck	1:10000	WB
HK2	2867	Cell Signaling Technology	1:1000/1:100	WB/IF
c-Myc	5605	Cell Signaling Technology	1:1000	WB
γH2AX	9718	Cell Signaling	1:1000/1:100	WB/IF
BrdU	ab6326	Abcam	1:200	IF
HIF-1	36169	Cell Signaling Technology	1:1000	WB
β-catenin	9562	Cell Signaling Technology	1:1000	WB
P-GSK3β	ab68476	Abcam	1:1000	WB
β-actin	A5316	Sigma	1:1000	WB
PFKFB3	ab181861	Abcam	1:1000	WB
LDHA	2012	Cell Signaling Technology	1:1000/1:100	WB/IF
LDHA	3582	Cell Signaling Technology	1:200	IHC
Peroxidase-conjugated secondary antibody anti-rabbit	77332	Sigma	1:10000	IF/WB
Donkey anti-Rabbit IgG (H+L) Highly cross-adsorbed secondary Antibody, Alexa Fluor™ 647	A31573	Invitrogen	1:200	IF
StarBright Blue 700 Fluorescent Secondary antibodies	12004160	Biorad	1:5000	WB

Supplementary Table 2: Primers list for mouse experiments

Primer Name	Forward (5'→3')	Reverse (5'→3')
PPIA	CATACAGGTCCTGGCATCTTGTC	AGACCACATGCTTGCCATCCAG
mMMP-1	AGGAAGGCGATATTGTGCTCTCC	TGGCTGGAAAGTGTGAGCAAGC
mMMP-2	CAAGGATGGACTCCTGGCACAT	TACTCGCCATCAGCGTTCCCAT
mMMP-9	GCTGACTACGATAAGGACGGCA	TAGTGGTGCAGGCAGAGTAGGA
mMMP-10	TGCTGCCTATGAGGCTCACAAC	GGAGGAAAACCGAGAGTGTGGA
mMMP-11	GATTGATGCTGCCTTCCAGGATG	CAGCGGAAAGTATTGGCAGGCT
mMMP-14	GGATGGACACAGAGAACTTCGTG	CGAGAGGTAGTTCTGGGTTGAG
mVEGF a	CTGCTGTAACGATGAAGCCCTG	GCTGTAGGAAGCTCATCTCTCC
Col1a2	TTCTGTGGGTCCTGCTGGGAAA	TTGTCACCTCGGATGCCTTGAG
Col6a1	GACACCTCTCAGTGTGCTCTGT	GCGATAAGCCTTGGCAGGAAATG
INF-γ	CAAGTGGCATAGATGTGGAAGA	GACGCTTATGTTGTTGCTGATG
TNFα	CCCACGTCGTAGCAAACCAC	GCAGCCTTGTCCCTTGAAGA
IL-6	CTCTGGGAAATCGTGGAAATG	AAGTGCATCATCGTTGTTCATACA
HPRT1	GGCCAGACTTTGTTGGATTTG	CGCTCATCTTAGGCTTTGTATTTG
RPL13	GTGGACACTTGGTTCAACCAGC	GGTGTGGTATCTCACTGTAGGG
LAMB 3	TGACCAGACCTATGGACACGTG	GTCACAGTGACCTCGTTGGCAT
F4/80	CTTTGGCTATGGGCTTCCAGTC	GCAAGGAGGACAGAGTTTATCGTG
CCL2	AGTAGGCTGGAGAGCTACAA	GTATGTCTGGACCCATTCCTTC

Supplementary Table 3: Antibodies used in the flow cytometry

Antibody Target	Color	Dilution	Supplier	Catalog number
CD3	BV510	1:100	BD Biosciences	740113
CD19	BV510	1:100	BD Biosciences	562956
CD45R	BV510	1:100	Biolegend	103248
Ly6g	BV510	1:100	BD Biosciences	740157
CD 11b	BV570	1:50	Biolegend	101233
CD 11c	BV650	1:200	Biolegend	117339
CD 16.2	BV421	1:100	Biolegend	149521
CD 26	BUV737	1:50	BD Biosciences	741729
CD43	BV786	1:100	BD Biosciences	740857
CD45	BUV395	1:200	BD Biosciences	564279
CD64	BV711	1:50	Biolegend	139311
CD209b	APC	1:100	Invitrogen	17209382
CD206	BV605	1:50	Biolegend	141721
CX3CR1	PacBlue	1:50	Biolegend	149038
F4/80	BUV805	1:50	BD Biosciences	749282
LYVE1	PE/Cy7	1:100	Invitrogen	25044382
LY6C	APC/Fire810	1:100	Biolegend	128055
MHCII	AF700	1:200	Biolegend	107621
TIM4	PerCP/eFluor710	1:100	Invitrogen	15579286
CD3	PECy5	1:100	BD Pharmingen	555276
CD4	APC Cy7	1:100	Biolegend	100414
CD8	BUV496	1:100	BD Biosciences	752636
CD44	BV605	1:100	Biolegend	103047
CD45	BUV 661	1:100	BD Biosciences	612975
CD62L	PerCP	1:100	Biolegend	104429
CD279	APC	1:100	Biolegend	109112
NK1.1	PE Fire810	1:100	Biolegend	108767
KI67	BUV395	1:100	BD Biosciences	564071
TNF-a	BV785	1:100	Biolegend	506341
CD11b	BV 421	1:50	Sony	1106180
F4/80	APC	1:50	Sony	1215575
MHC II	APC-Cy7	1:50	BD	107627
CD206	AF700	1:50	Sony	1308670

CD192 (CCR2)	APC Fire 750	1:50	Sony	1353150
CD11c	PECy7	1:50	BD	558075
CountBright ™ Absolute Counting Beads		10µL	ThermoFisherScientific	C36950
Fixable LIVE/DEAD ™ blue		1:1000	ThermoFisherScientific	L23105

Supplementary Table 4: Primary antibodies use for immunostaining experiments in human tissues

Antibody target	Supplier	Dilution
PDL1	PhenoCode	1:200
PD1	PhenoCode	1:75
CD68	PhenoCode	1:8000
FOXP3	PhenoCode	1:100
CD163	PhenoCode	1:600
CD8	PhenoCode	1:2000

Supplementary Table 5: Patient characteristics

Patient ID	Age (yrs.)	Sex	PROS Phenotype	Sample	Nucleotide Variation	Amino acid Variation	Variant Allele Frequency (%)
22NA10303	7	M	CLOVES	Skin	c.317G>T	p.G106V	23
23NA00925	12	M	CLOVES	Skin	c.3140A>G	p.H1047R	10
23NA05189	30	F	CLOVES	Skin	c.2176G>A	p.E726K	12
23NA05579	24	F	Klippel Trenaunay syndrome	Skin	c.1633G>A	p.E545K	6
23NA06000	64	F	Klippel Trenaunay syndrome	Skin	c.325_327delGAA	p.E109_E110del	2
23NA06045	2	F	CLOVES	Skin	c.1357G>A	p.E453K	18
23NA06519	51	F	CLOVES	Skin	c.317G>T	p.G106V	8
23NA10385	43	F	Isolated venous malformation	Skin	c.1638G>C	p.Q546H	2
24NA01153	36	M	CLOVES	Skin	c.3139C>T	p.H1047Y	17
24NA02622	1.5	M	FAVA	Skin	c.12589T>C	p.C420R	17

PROS: PIK3CA-Related Overgrowth Spectrum

CLOVES: Congenital Lipomatosis Overgrowth Vascular malformation Epidermal nevi and Skeletal anomalies

FAVA: Fibro Adipose Vascular Anomaly
