# Supplementary material for: Oncogenic PIK3CA mutations shape an immunoregulatory microenvironment in mosaic overgrowth disorders
Source: PNAS Nexus. 2026 May 13;5(6):pgag163. doi: 10.1093/pnasnexus/pgag163 (PMC13222027; doi:10.1093/pnasnexus/pgag163)
Supplement: pgag163_Supplementary_Data [file pgag163_supplementary_data.zip › PNASNEXUS-PNASNEXUS-2025-01923-TR-s03.pdf]

Supplementary Table 1: Primary and secondary antibodies used for Western blot and immunostaining in mouse experiments

Antibody target	Catalog Number	Supplier	Dilution	Application
p110α	4249	Cell Signaling Technology	1:1000	WB
P-AKT (Thr308)	13038	Cell Signaling Technology	1:1000	WB
PKM2	4053	Cell Signaling Technology	1:1000/1:100	WB/IF
P-S6RP	5364	Cell Signaling Technology	1:1000	WB
α-Tubulin	t5168	Merck	1:10000	WB
HK2	2867	Cell Signaling Technology	1:1000/1:100	WB/IF
c-Myc	5605	Cell Signaling Technology	1:1000	WB
γH2AX	9718	Cell Signaling	1:1000/1:100	WB/IF
BrdU	ab6326	Abcam	1:200	IF
HIF-1	36169	Cell Signaling Technology	1:1000	WB
β-catenin	9562	Cell Signaling Technology	1:1000	WB
P-GSK3β	ab68476	Abcam	1:1000	WB
β-actin	A5316	Sigma	1:1000	WB
PFKFB3	ab181861	Abcam	1:1000	WB
LDHA	2012	Cell Signaling Technology	1:1000/1:100	WB/IF
LDHA	3582	Cell Signaling Technology	1:200	IHC
Peroxidase-conjugated secondary antibody anti-rabbit	77332	Sigma	1:10000	IF/WB
Donkey anti-Rabbit IgG (H+L) Highly cross-adsorbed secondary Antibody, Alexa Fluor™ 647	A31573	Invitrogen	1:200	IF
StarBright Blue 700 Fluorescent Secondary antibodies	12004160	Biorad	1:5000	WB

Supplementary Table 2: Primers list for mouse experiments

Primer Name	Forward (5'→3')	Reverse (5'→3')
PPIA	CATACAGGTCCTGGCATCTTGTC	AGACCACATGCTTGCCATCCAG
mMMP-1	AGGAAGGCGATATTGTGCTCTCC	TGGCTGGAAAGTGTGAGCAAGC
mMMP-2	CAAGGATGGACTCCTGGCACAT	TACTCGCCATCAGCGTTCCCAT
mMMP-9	GCTGACTACGATAAGGACGGCA	TAGTGGTGCAGGCAGAGTAGGA
mMMP-10	TGCTGCCTATGAGGCTCACAAC	GGAGGAAAACCGAGAGTGTGGA
mMMP-11	GATTGATGCTGCCTTCCAGGATG	CAGCGGAAAGTATTGGCAGGCT
mMMP-14	GGATGGACACAGAGAACTTCGTG	CGAGAGGTAGTTCTGGGTTGAG
mVEGF a	CTGCTGTAACGATGAAGCCCTG	GCTGTAGGAAGCTCATCTCTCC
Col1a2	TTCTGTGGGTCCTGCTGGGAAA	TTGTCACCTCGGATGCCTTGAG
Col6a1	GACACCTCTCAGTGTGCTCTGT	GCGATAAGCCTTGGCAGGAAATG
INF-γ	CAAGTGGCATAGATGTGGAAGA	GACGCTTATGTTGTTGCTGATG
TNFα	CCCACGTCGTAGCAAACCAC	GCAGCCTTGTCCCTTGAAGA
IL-6	CTCTGGGAAATCGTGGAAATG	AAGTGCATCATCGTTGTTCATACA
HPRT1	GGCCAGACTTTGTTGGATTTG	CGCTCATCTTAGGCTTTGTATTTG
RPL13	GTGGACACTTGGTTCAACCAGC	GGTGTGGTATCTCACTGTAGGG
LAMB 3	TGACCAGACCTATGGACACGTG	GTCACAGTGACCTCGTTGGCAT
F4/80	CTTTGGCTATGGGCTTCCAGTC	GCAAGGAGGACAGAGTTTATCGTG
CCL2	AGTAGGCTGGAGAGCTACAA	GTATGTCTGGACCCATTCCTTC

Supplementary Table 3: Antibodies used in the flow cytometry

Antibody Target	Color	Dilution	Supplier	Catalog number
CD3	BV510	1:100	BD Biosciences	740113
CD19	BV510	1:100	BD Biosciences	562956
CD45R	BV510	1:100	Biolegend	103248
Ly6g	BV510	1:100	BD Biosciences	740157
CD 11b	BV570	1:50	Biolegend	101233
CD 11c	BV650	1:200	Biolegend	117339
CD 16.2	BV421	1:100	Biolegend	149521
CD 26	BUV737	1:50	BD Biosciences	741729
CD43	BV786	1:100	BD Biosciences	740857
CD45	BUV395	1:200	BD Biosciences	564279
CD64	BV711	1:50	Biolegend	139311
CD209b	APC	1:100	Invitrogen	17209382
CD206	BV605	1:50	Biolegend	141721
CX3CR1	PacBlue	1:50	Biolegend	149038
F4/80	BUV805	1:50	BD Biosciences	749282
LYVE1	PE/Cy7	1:100	Invitrogen	25044382
LY6C	APC/Fire810	1:100	Biolegend	128055
MHCII	AF700	1:200	Biolegend	107621
TIM4	PerCP/eFluor710	1:100	Invitrogen	15579286
CD3	PECy5	1:100	BD Pharmingen	555276
CD4	APC Cy7	1:100	Biolegend	100414
CD8	BUV496	1:100	BD Biosciences	752636
CD44	BV605	1:100	Biolegend	103047
CD45	BUV 661	1:100	BD Biosciences	612975
CD62L	PerCP	1:100	Biolegend	104429
CD279	APC	1:100	Biolegend	109112
NK1.1	PE Fire810	1:100	Biolegend	108767
KI67	BUV395	1:100	BD Biosciences	564071
TNF-a	BV785	1:100	Biolegend	506341
CD11b	BV 421	1:50	Sony	1106180
F4/80	APC	1:50	Sony	1215575
MHC II	APC-Cy7	1:50	BD	107627
CD206	AF700	1:50	Sony	1308670

CD192 (CCR2)	APC Fire 750	1:50	Sony	1353150
CD11c	PECy7	1:50	BD	558075
CountBright ™ Absolute Counting Beads		10µL	ThermoFisherScientific	C36950
Fixable LIVE/DEAD ™ blue		1:1000	ThermoFisherScientific	L23105

Supplementary Table 4: Primary antibodies use for immunostaining experiments in human tissues

Antibody target	Supplier	Dilution
PDL1	PhenoCode	1:200
PD1	PhenoCode	1:75
CD68	PhenoCode	1:8000
FOXP3	PhenoCode	1:100
CD163	PhenoCode	1:600
CD8	PhenoCode	1:2000

Supplementary Table 5: Patient characteristics

Patient ID	Age (yrs.)	Sex	PROS Phenotype	Sample	Nucleotide Variation	Amino acid Variation	Variant Allele Frequency (%)
22NA10303	7	M	CLOVES	Skin	c.317G>T	p.G106V	23
23NA00925	12	M	CLOVES	Skin	c.3140A>G	p.H1047R	10
23NA05189	30	F	CLOVES	Skin	c.2176G>A	p.E726K	12
23NA05579	24	F	Klippel Trenaunay syndrome	Skin	c.1633G>A	p.E545K	6
23NA06000	64	F	Klippel Trenaunay syndrome	Skin	c.325_327delGAA	p.E109_E110del	2
23NA06045	2	F	CLOVES	Skin	c.1357G>A	p.E453K	18
23NA06519	51	F	CLOVES	Skin	c.317G>T	p.G106V	8
23NA10385	43	F	Isolated venous malformation	Skin	c.1638G>C	p.Q546H	2
24NA01153	36	M	CLOVES	Skin	c.3139C>T	p.H1047Y	17
24NA02622	1.5	M	FAVA	Skin	c.12589T>C	p.C420R	17

PROS: PIK3CA-Related Overgrowth Spectrum

CLOVES: Congenital Lipomatosis Overgrowth Vascular malformation Epidermal nevi and Skeletal anomalies

FAVA: Fibro Adipose Vascular Anomaly

Supplementary Figure 1: Activating mutation in PIK3CA drives a metabolic shift towards a Warburg-like phenotype. (**A**) Western blot for HK2, PKM2, and LDHA of primary fibroblasts kept in low glucose conditions derived from PIK3CA^{WT}, PIK3CA$^{CAGG\text{-}CreER,}$ and PIK3CA^{HO} mice (n = 3 per group). (**B**) Quantification. (**C**) Representative immunofluorescence of mitochondrial state and organization using mitotracker in PIK3CA^{WT} and PIK3CA$^{CAGG\text{-}CreER}$ derived primary fibroblasts. Images were taken using spinning disk, scale bar 10 μm. (**D**) Analysis of the structure and mitochondrial organization of the images taken using the spinning disk. We used the ImageJ plugin MINA to calculate mitochondrial footprint, branch length, and network branches (n = 5 per condition). (**E**) Flow cytometry quantification of the geometric mean of TMRE (membrane potential) and Mitotracker (mitochondrial mass) in PIK3CA^{WT} and PIK3CA$^{CAGG\text{-}CreER}$ fibroblasts (n = 3 per condition) cultured in low-glucose conditions. (**F**) Oxygen consumption rate (OCR) was measured using an extracellular flux analyser (Seahorse Bioscience) in PIK3CA^{WT} and PIK3CA$^{CAGG\text{-}CreER}$ fibroblasts (n = 3 per condition). (**G**) Overview of the enriched metabolites (top 25) inferred from the metabolic experiment in PIK3CA^{WT} and PIK3CA$^{CAGG\text{-}CreER}$ primary fibroblasts in low glucose condition. (**H**) Volcano plot of the enriched metabolites of the same experiment. (**I**) Western blot and quantification of PFKFB3 in primary fibroblasts kept in low glucose conditions derived from PIK3CA^{WT} and PIK3CA$^{CAGG\text{-}CreER}$ mice (n = 9 per group).

Supplementary Figure 2: Metabolite changes observed in fibroblasts derived from PIK3CA$^{CAGG\text{-}CreER}$ mice. Graphic example of metabolite modifications observed (n = 3 per condition). AU: Arbitrary units.

Supplementary Figure 3: Metabolite changes observed in fibroblasts derived from PIK3CA$^{CAGG\text{-}CreER}$ mice. Graphic example of metabolite modifications observed (n = 3 per condition). AU: Arbitrary units.

Supplementary Figure 4: PIK3CA mutation is associated with ROS accumulation. (**A**) Representative immunofluorescence of HIF-1 in primary fibroblasts derived from PIK3CA^{WT} and PIK3CA$^{CAGG\text{-}CreER}$ mice cultured in low glucose conditions and quantification. (**B**) Western blot and quantification of the components of β-catenin/GSK3-β pathway (P-GSK3-β, β-catenin, c-Myc, P-AKT, P-S6RP). Cells were treated with Laduviglusib 3μM, GSK3- β inhibitor for three hours (n = 3 per group). (**C**) Graphical representation of the interaction between AKT and β-catenin/GSK3- β pathway. (**D**) The ratio between Glutathione disulfide (GSSG) and Glutathione (GSH)

inferred by the different metabolic experiments that we performed (n= 9 per condition). (**E**) Raw data of the flow cytometry experiment to calculate ROS production of data is shown in Figure 1H. In green are represented the wild type in blue the mutated ones in terms of area of positive cells.

Supplementary Figure 5: PIK3CA mutation is associated with PH2AX accumulation. (**A**) Representative Immunofluorescence of PH2AX in primary fibroblasts derived from $PIK3CA^{WT}$ and $PIK3CA^{CAGG\text{-}CreER}$ mice with quantification. (**B**) Representative immunohistochemistry of PH2AX in the liver of $PIK3CA^{WT}$ and $PIK3CA^{CAGG\text{-}CreER}$ mice. (**C**) BrdU staining and quantification in the liver of $PIK3CA^{WT}$ and $PIK3CA^{CAGG\text{-}CreER}$ mice (n= 3 per group). (**D**) Western blot and quantification of HK2, PKM2 and LDHA in primary fibroblasts derived from $PIK3CA^{WT}$ and $PIK3CA^{CAGG\text{-}CreER}$ mice (n = 6 per group). Cells were treated as indicated in the figure with Alpelisib 1 µM or DMSO during 24h.

Supplementary Figure 6: PIK3CA mutations are associated with enhanced infiltration of liver resident macrophages. (A) Gating strategy for the macrophage infiltration in the liver of $PIK3CA^{WT}$ and $PIK3CA^{CAGG\text{-}CreER}$ mice. (**B**) Gating strategy for primary fibroblasts derived from $PIK3CA^{WT}$ and $PIK3CA^{CAGG\text{-}CreER}$ mice, used for the quantifications shown in Figure 2C.

Supplementary Figure 7: PIK3CA mutations are associated with enhanced infiltration of adipose resident macrophages. (A) Gating strategy for the macrophage infiltration in the adipose tissue of $Adipo^{WT}$ and $Adipo^{CreER}$ mice. (**B**) Cluster proportions in $PIK3CA^{WT}$ and $PIK3CA^{CAGG\text{-}CreER}$ mice shown in Figure 3D. (**C**) Percentages of population for clusters derived from the single cell experiment for Figure 3E. (**D**) IL-1β, Il-2 and IL-4 measurements in serum derived from $Adipo^{WT}$ (n= 5-6) and $Adipo^{CreER}$ mice (n=6).

Supplementary Figure 8: Matrix remodelling and increase in fibrosis in tissues bearing PIK3CA overactivating mutation. (A) Masson's Trichrome staining of the liver of $PIK3CA^{WT}$ and $PIK3CA^{CAGGCreER}$ mice six weeks after tamoxifen induction. (**B**) Masson's Trichrome staining of adipose tissue of $Adipo^{WT}$ and $Adipo^{CreE}$ six weeks after tamoxifen induction. (**C**) mRNA expression of different components of the extracellular matrix of the adipose tissue of $Adipo^{WT}$ and $Adipo^{CreE}$ mice (n= 3-6 mice per group).

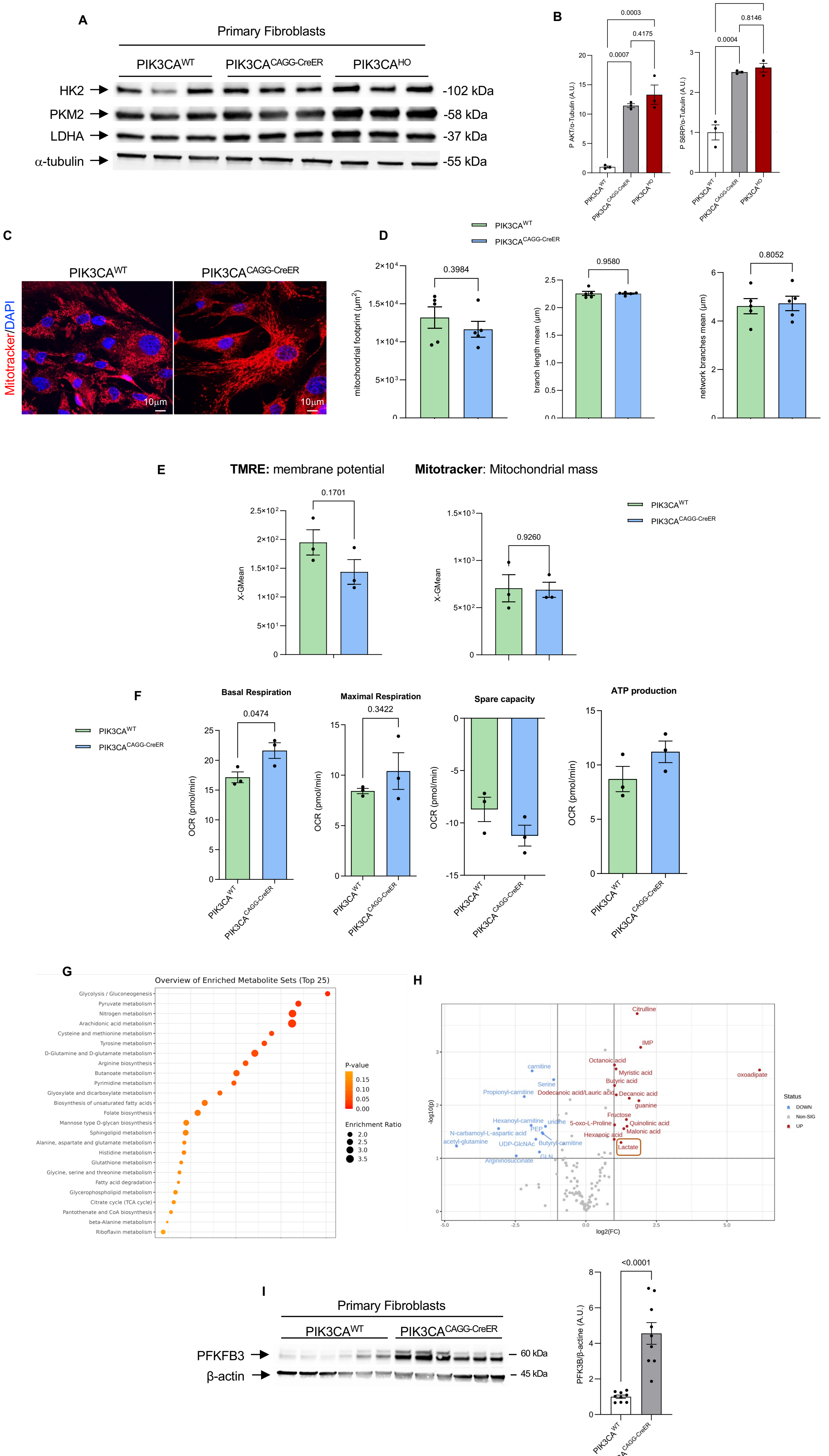

Supplementary Figure 1

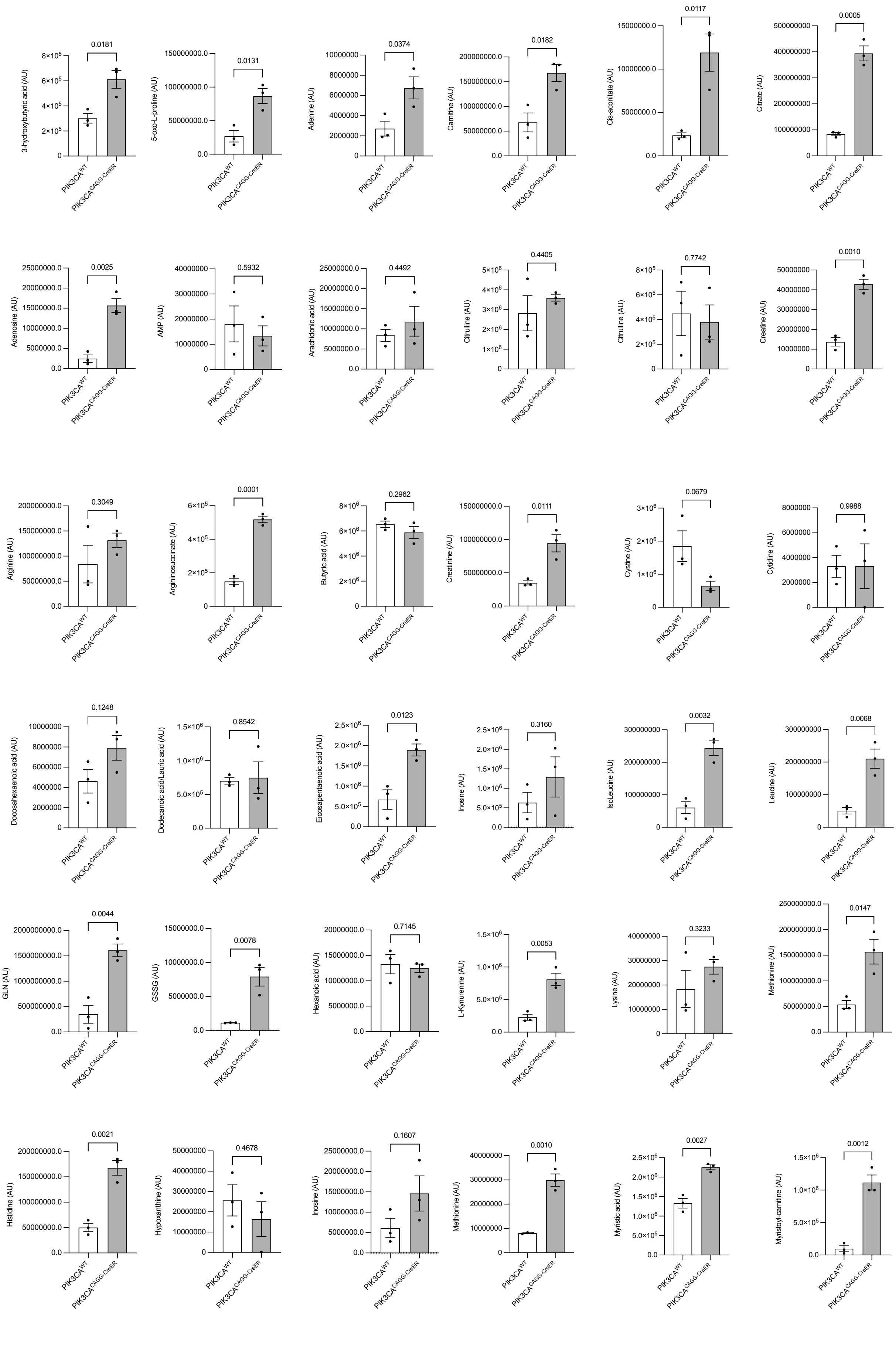

Supplementary Figure 2

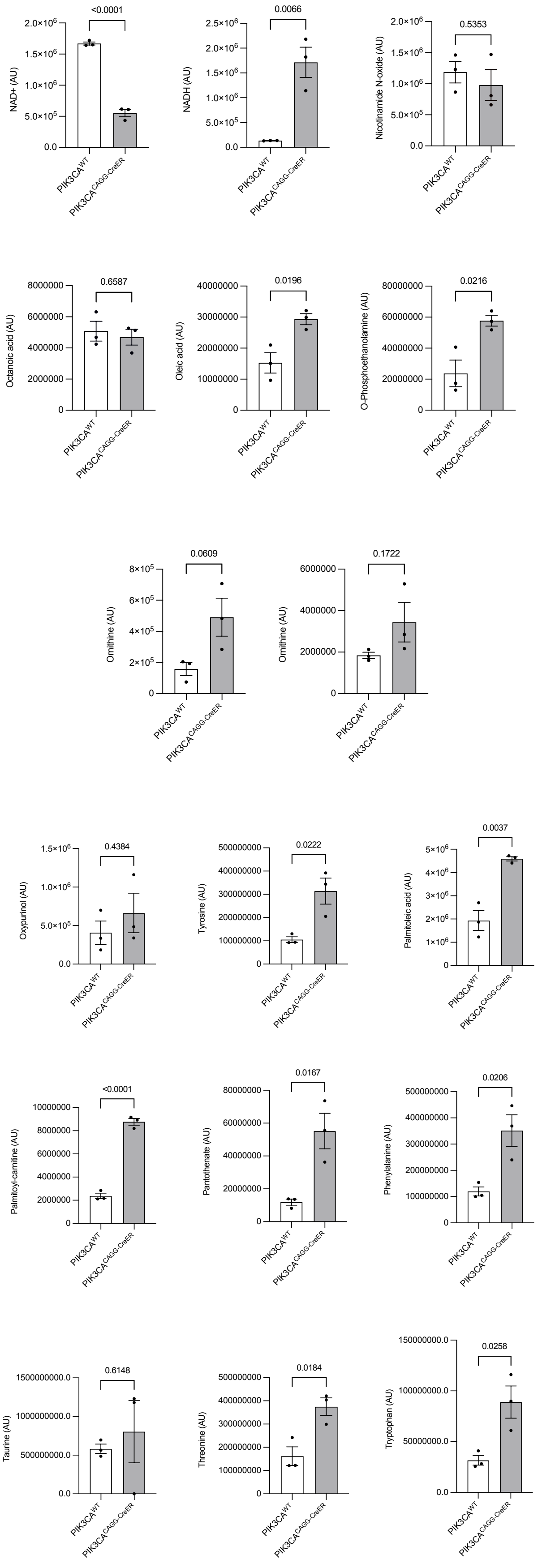

Supplementary Figure 3

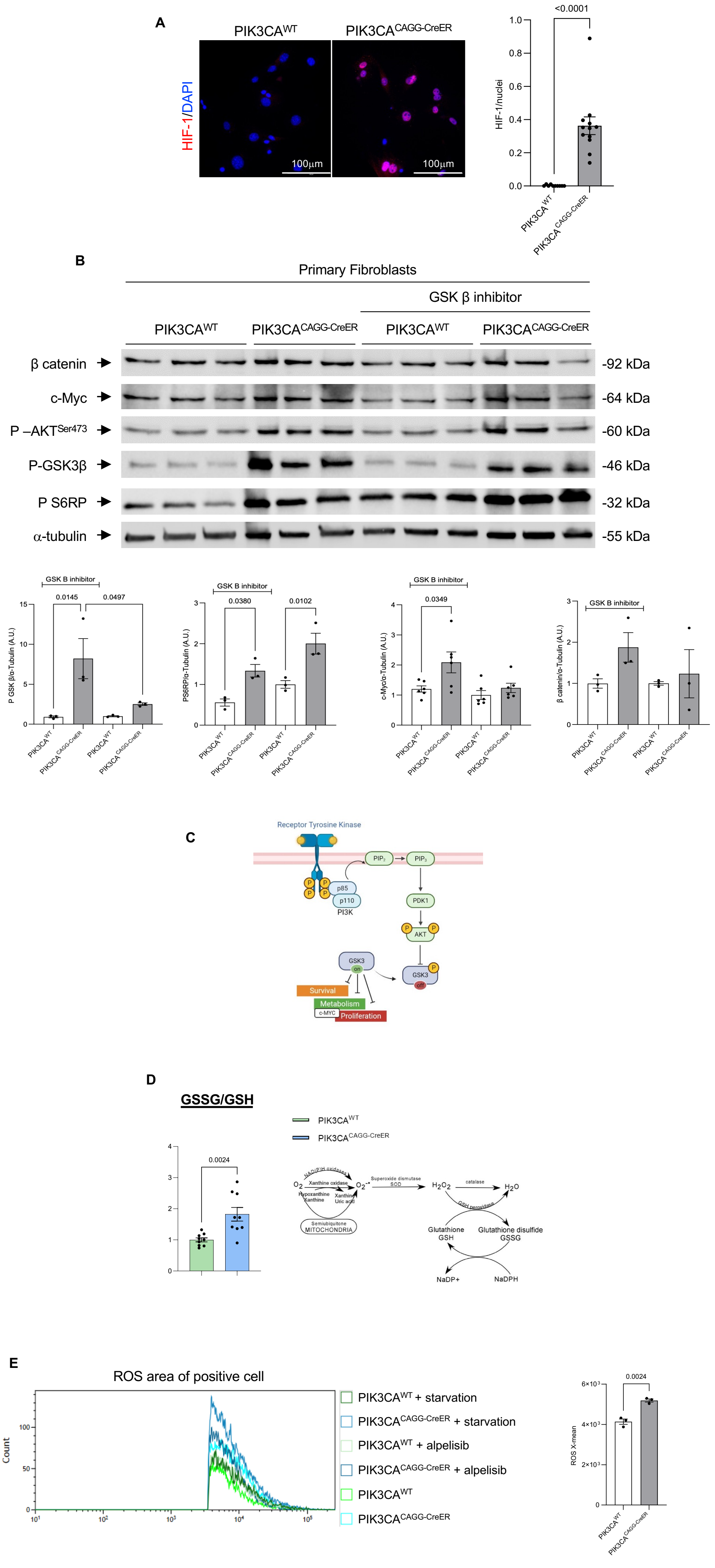

Supplementary Figure 4

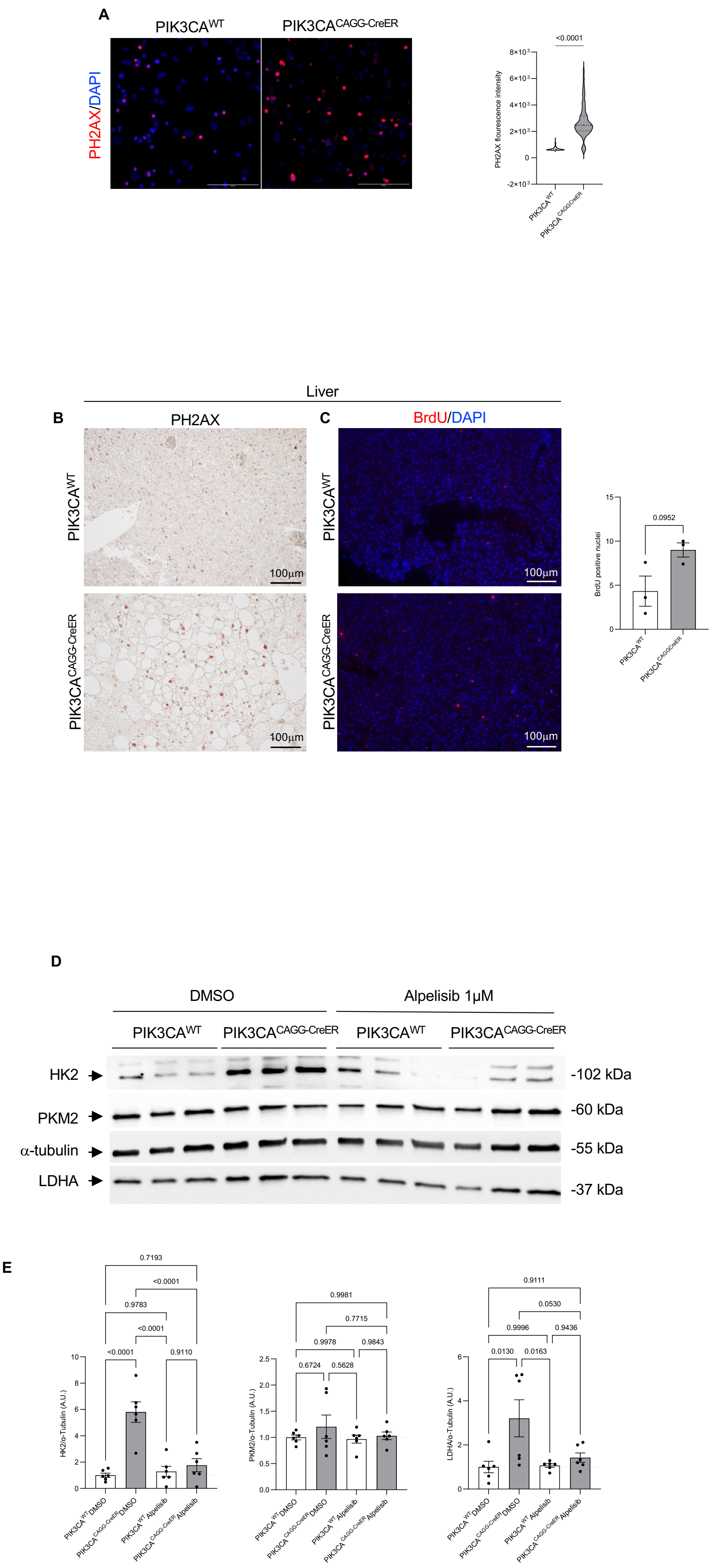

Supplementary Figure 5

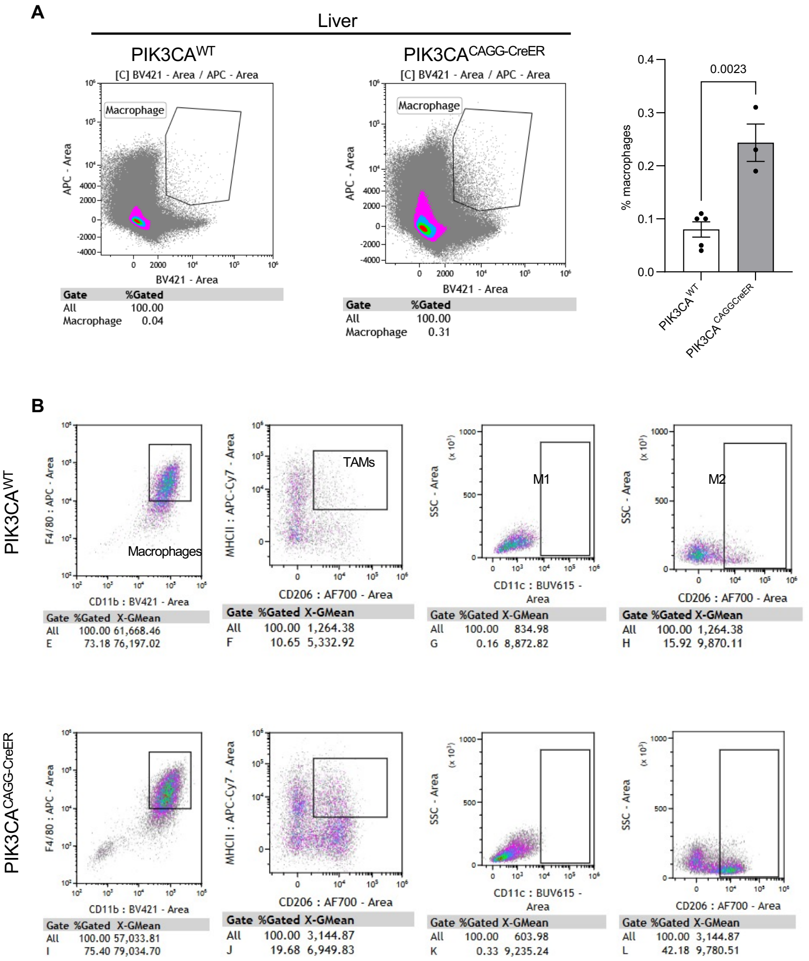

Supplementary Figure 6

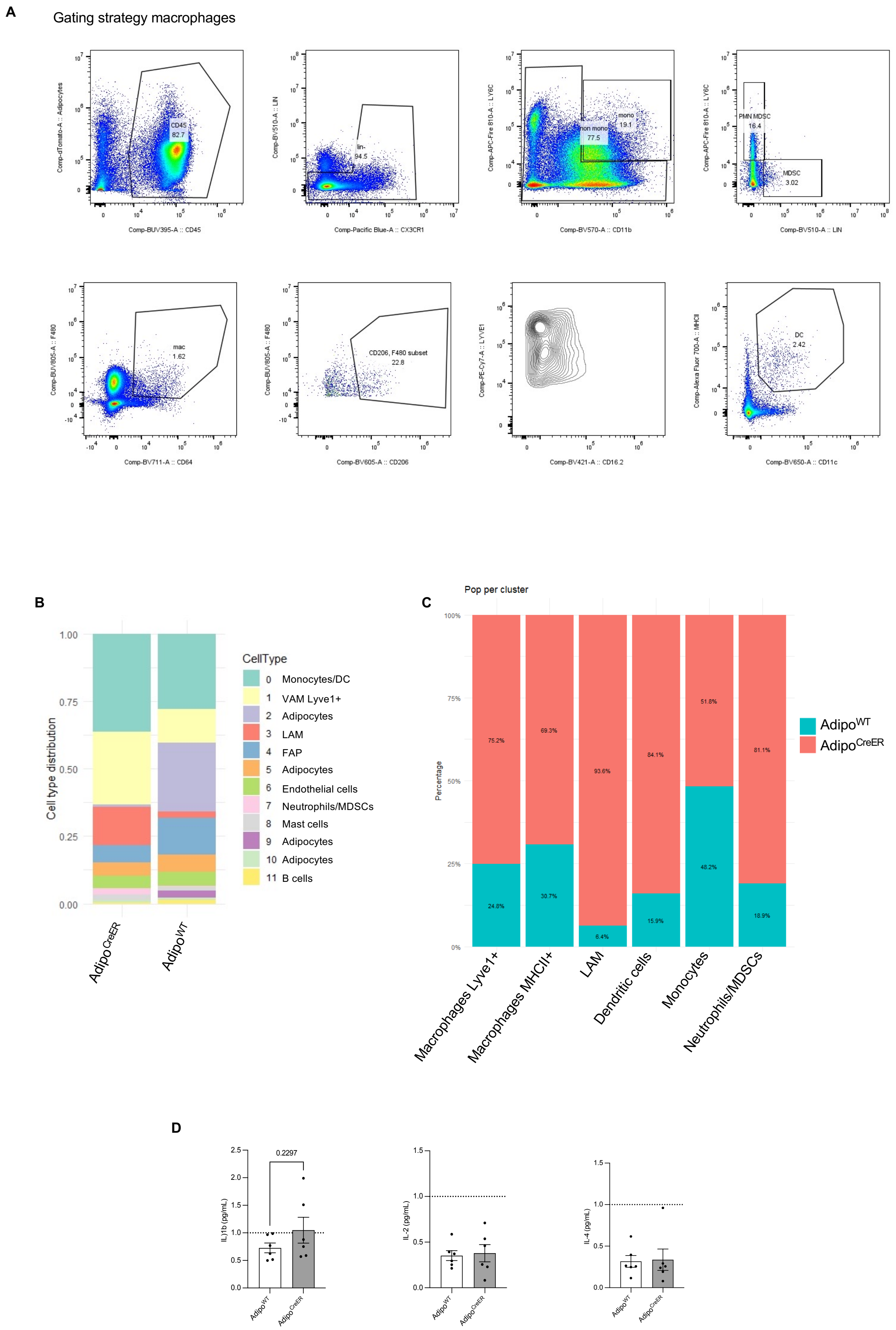

Supplementary Figure 7

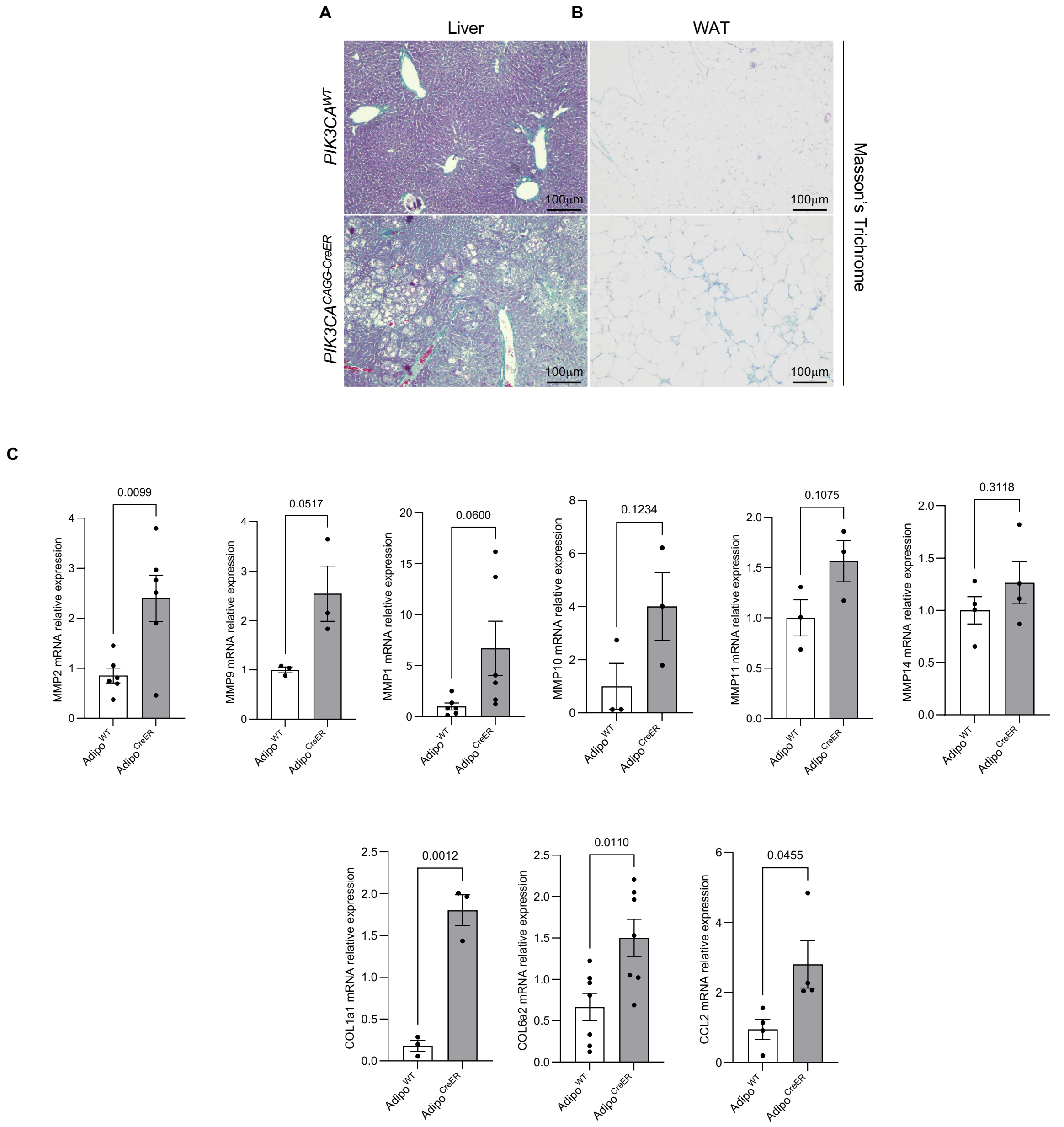

Supplementary Figure 8
