# Supplementary figures and images for: Oncogenic PIK3CA mutations shape an immunoregulatory microenvironment in mosaic overgrowth disorders
Source: PNAS Nexus. 2026 May 13;5(6):pgag163. doi: 10.1093/pnasnexus/pgag163 (PMC13222027; doi:10.1093/pnasnexus/pgag163)

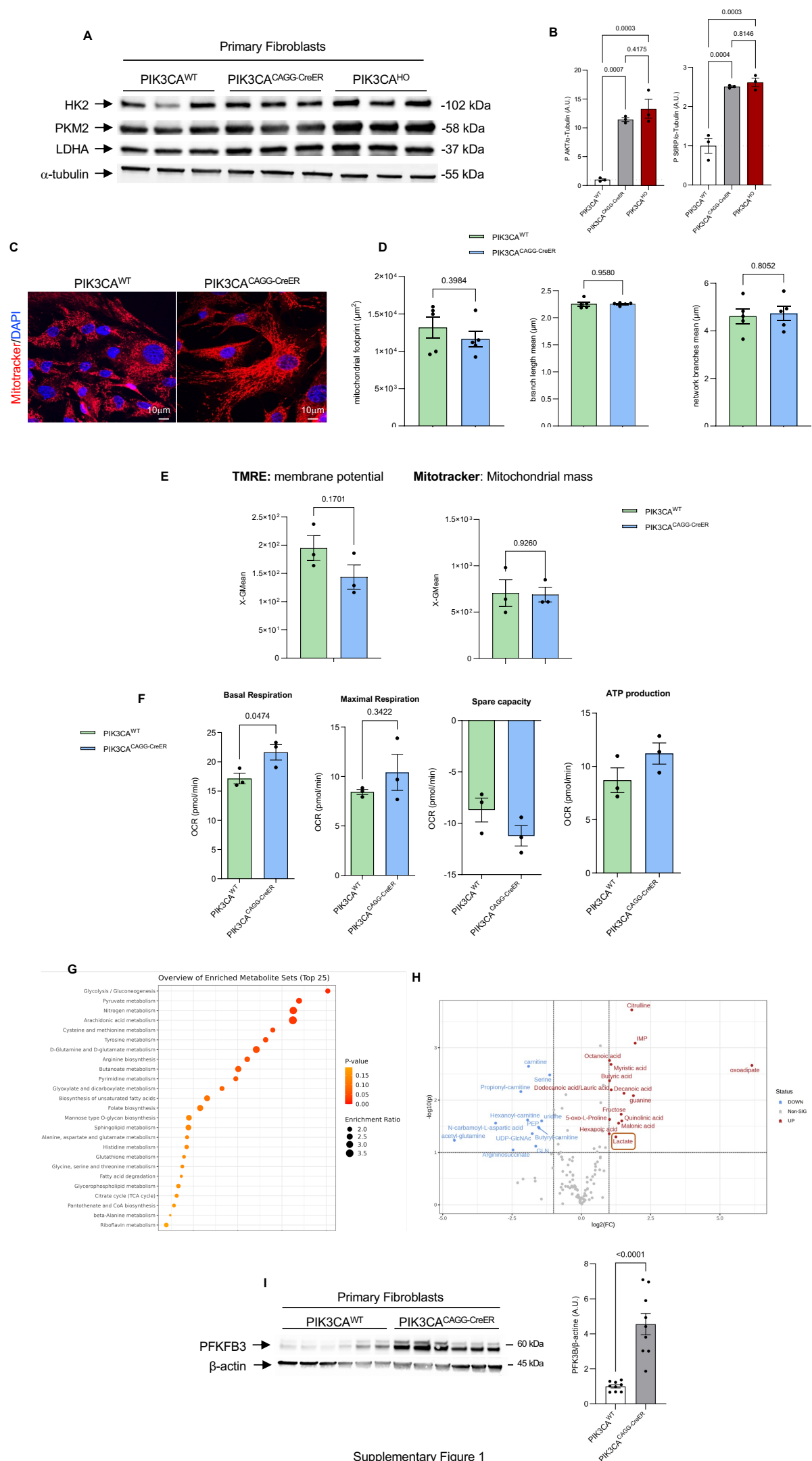

Supplementary Figure 1

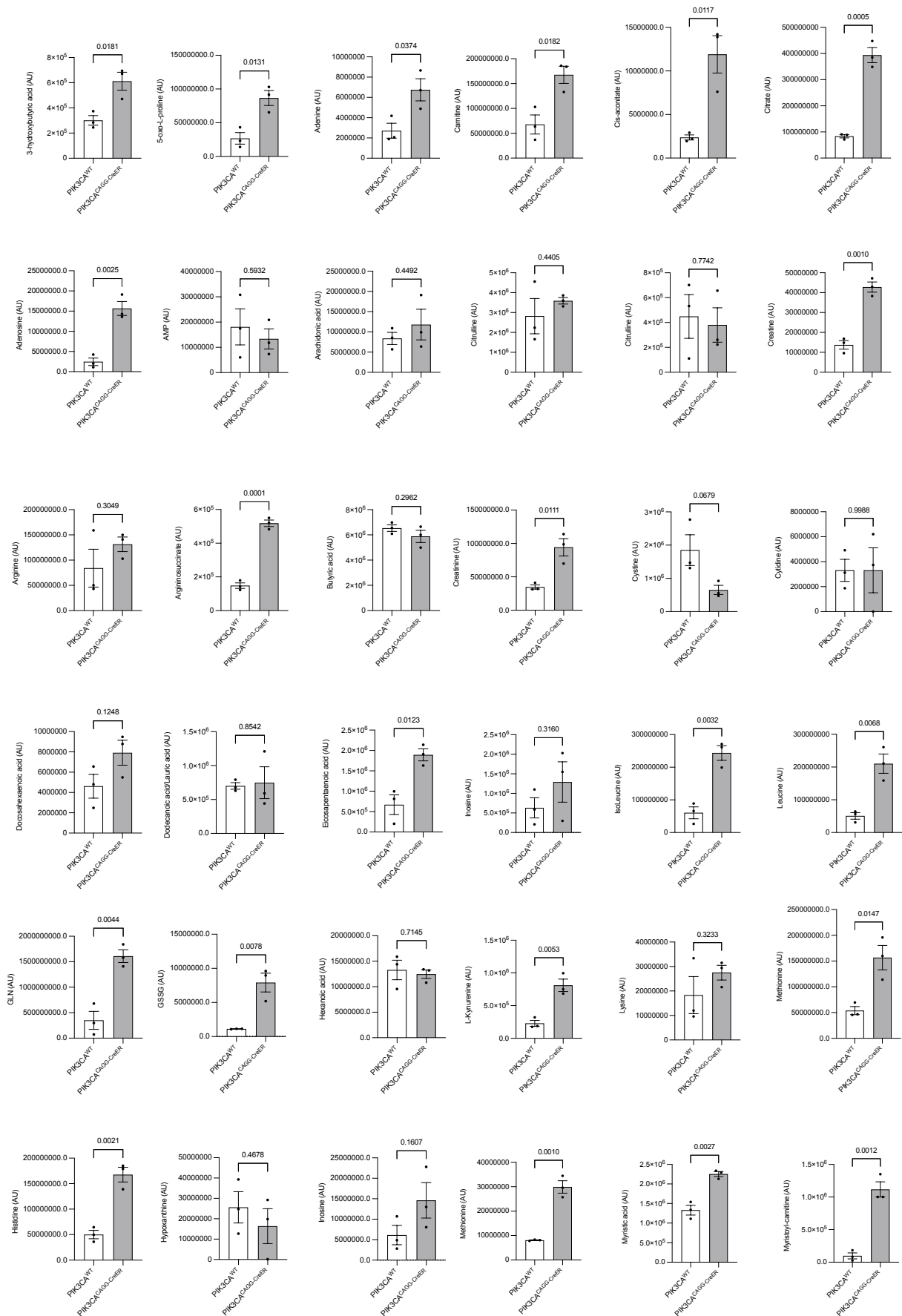

Supplementary Figure 2



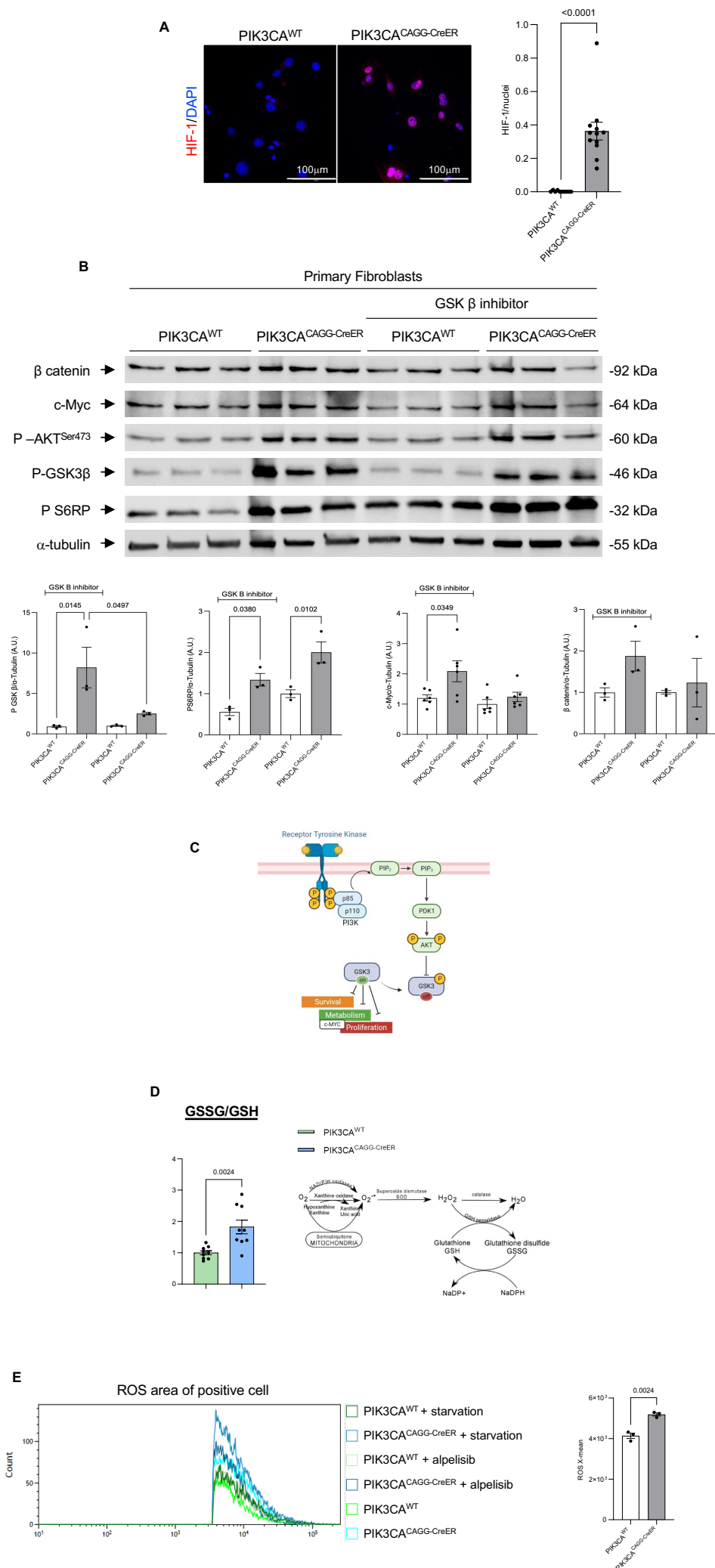

Supplementary Figure 4



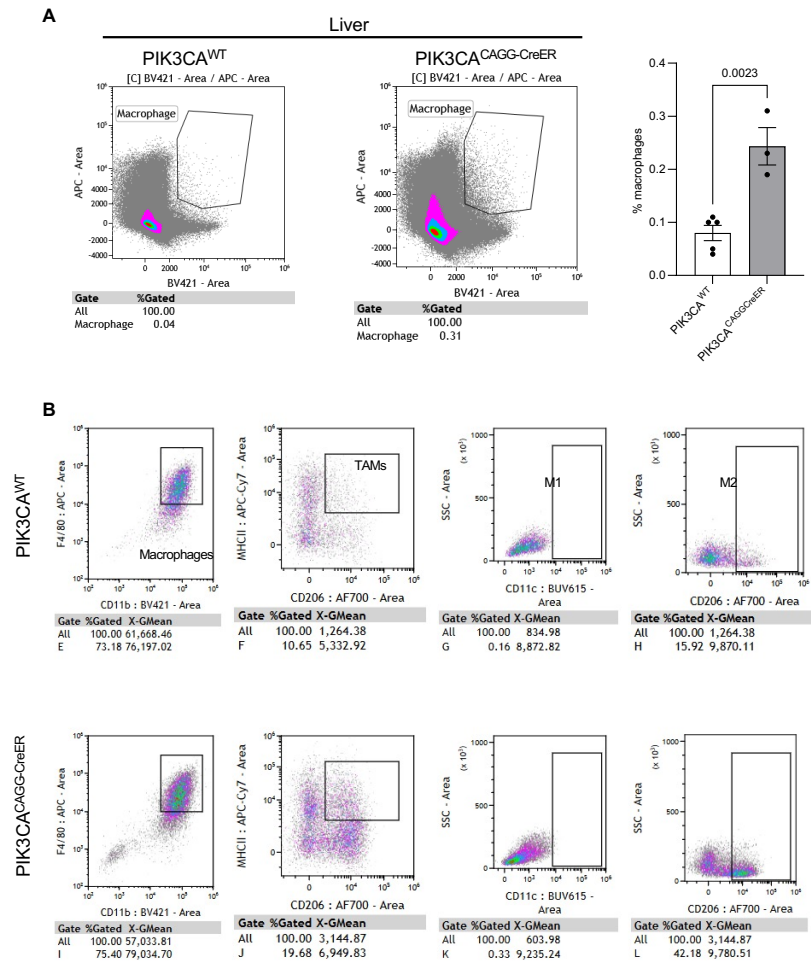

**A**

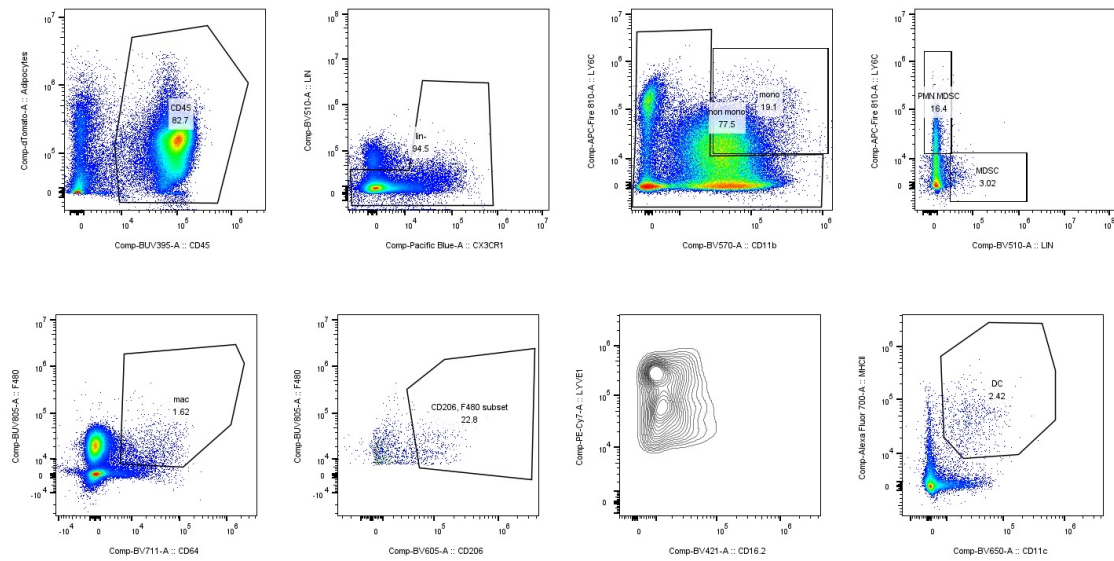

**B**

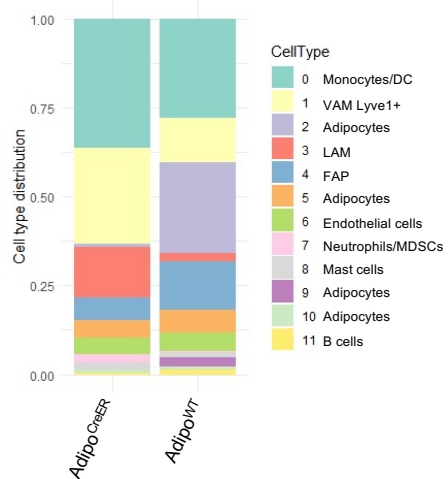

**C**

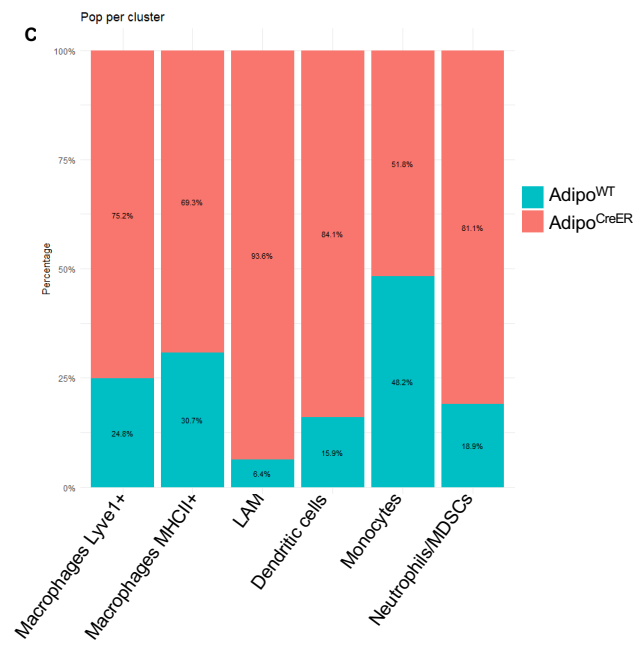

## D

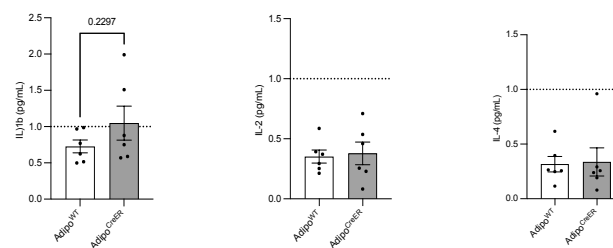

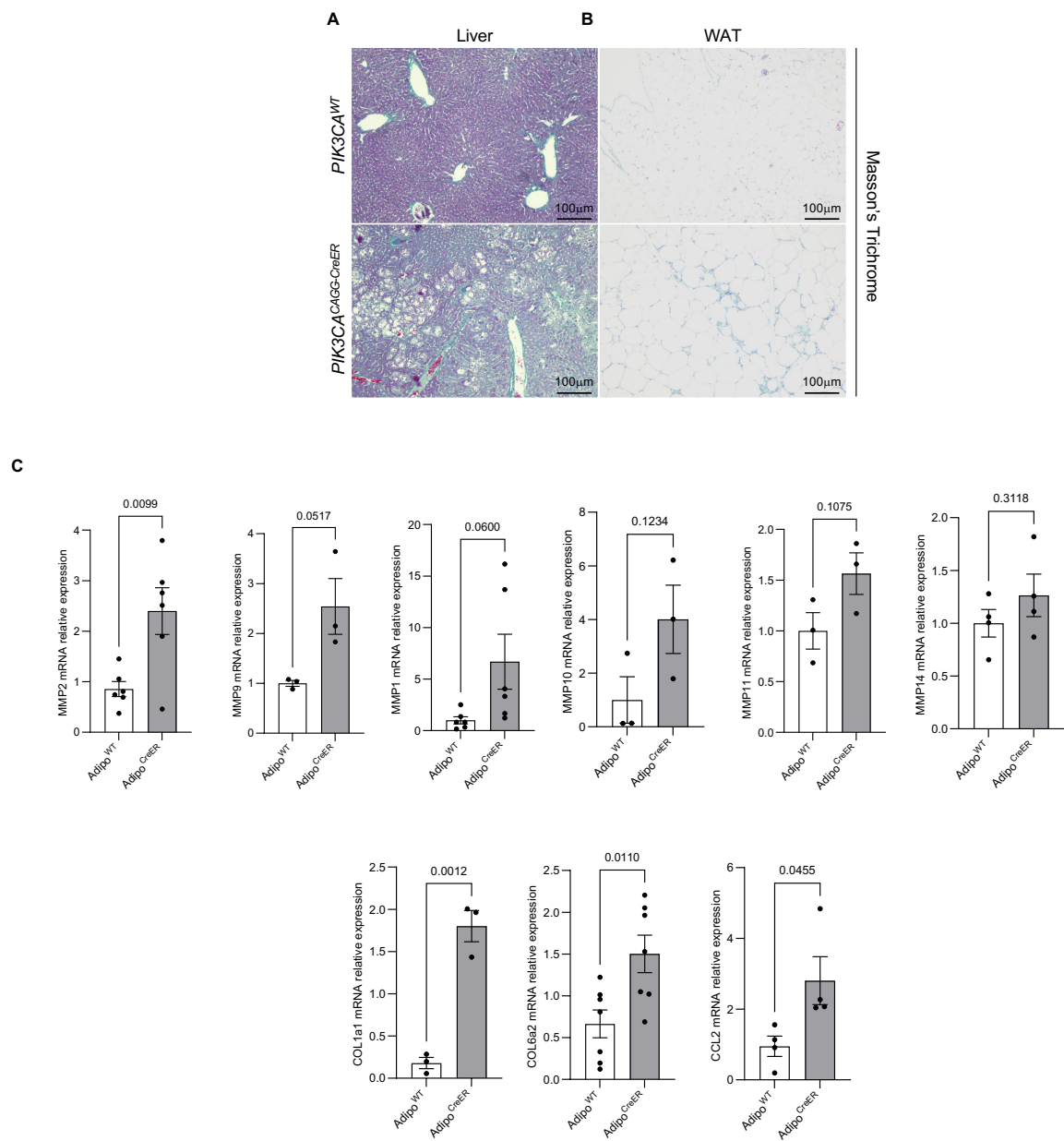

Supplement: pgag163_Supplementary_Data [file pgag163_supplementary_data.zip › PNASNEXUS-PNASNEXUS-2025-01923-TR-s01.pdf]
